# Congenital Hypothyroidism Dysregulates TRPC6 to Mediate Abnormal Dendritic Spine Growth of Hippocampal Neurons

**DOI:** 10.1111/cns.70618

**Published:** 2025-09-25

**Authors:** Tianci Li, Feifei Shen, Lingling Li, Peng Chen, Zhiwen Zhu, Yuqin Zheng, Haiying Li, Guihai Suo, Yongjun Wang, Jinlong Shi, Youjia Wu

**Affiliations:** ^1^ Department of Pediatrics Affiliated Hospital of Nantong University Nantong Jiangsu Province China; ^2^ Key Laboratory of Neuroregeneration of Jiangsu and Ministry of Education, co‐Innovation Center of Neuroregeneration Nantong University Nantong Jiangsu Province China; ^3^ Department of Neurosurgery Affiliated Hospital of Nantong University Nantong Jiangsu Province China

**Keywords:** CaMKIV, cognition, congenital hypothyroidism, hippocampus, TRPC6

## Abstract

**Background:**

Congenital hypothyroidism (CH) may lead to irreversible neurological dysfunction of offspring by affecting hippocampal morphogenesis. The dentate gyrus (DG) is primarily the affected tissue by the CH, in which the dendritic spine density of gyrus granule cells (DGCs) is significantly reduced, thereby resulting in the cognitive impairment. The CaMKIV/CREB signaling pathway has been shown to mediate the deficient growth of DGCs dendritic spines, but the mechanism of CH in modulating the Ca^2+^‐dependent CaMKIV is still elusive.

**Methods:**

CH model of rat pups was prepared by the supply of 0.02% methimazole in the drinking water of pregnant dams from the 9th day of gestation. The dendritic spine density of hippocampal DGCs was detected by Golgi staining before or after administration of drugs. Additionally, the expression of CH‐mediated effectors in the hippocampus or primary neurons was determined by Western blot or RT‐PCR, and the immunofluorescence or subcellular fractionation was used to examine the distribution of these factors.

**Results:**

In the present study, the calcium influx‐related channel TRPC6 was identified under the regulation of T3, which was significantly downregulated in the DGCs of CH pups. TRPC6 deficiency has been revealed to decrease the dendritic spine density by affecting intracellular calcium transients and the CaMKIV/CREB signaling pathway. Pharmacological activation of TRPC6 with hyperforin was shown to be efficient in the rescue of DGCs dendritic spines and in improving the cognitive function of CH pups.

**Conclusions:**

CH of the neonates leads to downregulation of TRPC6 in hippocampal dentate gyrus neurons, which affects calcium influx and decreases activation of CaMKIV and downstream signaling, thereby causing abnormal growth of DGCs' dendritic spines and impaired cognitive function in the offspring. This study provides a new target for CH‐mediated developmental abnormality of the hippocampus in the offspring.

## Graphical abstract

T3 loss in congenital hypothyroidism (CH) downregulates TRPC6 in dentate granule cells (DGCs), which reduces dendritic spine density via the CaMKIV/CREB signaling pathway. TRPC6 agonist hyperforin rescues DGCs' dendritic spines and cognitive function of CH pups.

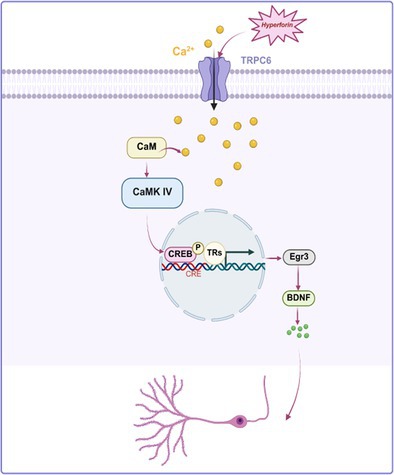

## 1 Introduction

Congenital hypothyroidism (CH) is a common neonatal disorder of the endocrine system with severe impacts on physical growth and neurodevelopment [1]. Unlike the reversible effects of thyroid hormone (TH) deficiency on abnormal metabolism and growth, CH may cause irreversible brain damage leading to mental retardation and impaired motor function [2, 3]. CH usually originates from defective thyroid gland development or insufficient synthesis and secretion of thyroxine [4]. But it can be caused by maternal factors, as the TH in the fetus is entirely supplied from the placenta before the end of the first trimester [5, 6]. The maternal thyroid disorders may induce intrauterine hypothyroidism, which in turn bears the risk of severe consequences including the lower birth weight and impairments of the infant's psychomotor and cognitive abilities [7, 8, 9]. Accumulative evidence has shown that the hippocampus is the primarily affected tissue by the CH [10]. Fetal and neonatal TH deficiency will give rise to the reduced synaptogenesis and connectivity, delayed myelin formation, disrupted axonal projections, and altered neurotransmitter levels in the hippocampus, leading to impaired organization and cognitive function [11, 12]. In the hippocampal dentate gyrus (DG) of CH pups, for example, the dendritic spine density of gyrus granule cells (DGCs) is markedly reduced, which is closely associated with cognitive deficits of the offspring [13]. Despite the regulatory mechanisms of CH‐mediated dysplasia of the hippocampus having been extensively investigated, they are still far beyond clear elucidation.

Ca^2+^ homeostasis is essential for normal cellular function in the brain [14]. Upon influx into neurons via plasma membrane receptors and voltage‐dependent ion channels [15], Ca^2+^ triggers cascades that drive gene transcription, protein synthesis, and cytoskeletal remodeling, thereby affecting synaptogenesis and synaptic transmission [16]. Calcium/calmodulin‐dependent protein kinase IV (CaMKIV) is one of the Ca^2+^‐response nuclear serine/threonine kinases implicated in regulating the dendrite growth of both cortical and hippocampal neurons via CREB signaling [17, 18]. CaMKIV deficiency has a severe impact on the brain development through decreasing dendrite complexity [19, 20]. Thus, CaMKIV is recognized as an important protein kinase to be required for cognitive functioning and other mental disorders [21]. Intriguingly, our previous works have uncovered that CH persistently suppresses the CaMKIV/CREB signaling pathway in hippocampal DGCs. CH‐mediated deficiency of CaMKIV in the DGCs results in the downregulation of early growth response factor 3 (EGR‐3), causing an insufficient production of neurotrophic factor BDNF. As a consequence, the density of dendritic spines is significantly reduced, and the cognitive function of the offspring is remarkably impaired [22, 23]. However, the mechanism of CH‐regulated CaMKIV deficiency has not been clarified so far.

Canonical transient receptor potential (TRPC) channels are the major calcium channels expressed in neurons and other cell types including thyroid cells, germ cells, and cardiomyocytes [24, 25, 26]. To date, seven TRPC (TRPC1‐7) channels have been identified, contributing to the pathogenesis of multiple diseases, especially to the neurological disorders such as autism, bipolar disorder (BD), and mental retardation [27]. Among these channels, TRPC1, TRPC2, and TRPC4 have been defined as triiodothyronine (T3)‐dependent Ca^2+^ channels important for evoking intracellular calcium signals and the development and maturation of neurons [28, 29, 30]. TRPC6 is abundantly expressed in the various regions of the brain. As a regulator of Ca^2+^ influx, TRPC6 mediates the survival of neurons, plasticity of the synapse, morphological changes of spines, and the length of neurites [31, 32, 33]. TRPC6 deficiency suppresses the phosphorylation of Ca^2+^‐sensitive kinases and impairs the dendritic growth of hippocampal neurons [33, 34, 35]. Given the physiological importance of the TRPC channels in response to T3‐mediated development of the neural circuit and in evoking intracellular calcium transients, it is assumed that TRPC6, the essential component of excitatory synapses, is the critical player in CH‐mediated dendritic spine density deficiency in DGCs through regulating the CaMKIV/EGR3/BDNF axis. In the present, we established a CH model of rat fetus and examined TRPC6 expression changes in the DGCs. TRPC6 deficiency in the DGCs was revealed to impair DGC dendrite growth by inactivation of the CaMKIV/EGR3/BDNF axis. The TRPC6 agonist hyperforin was shown to be efficient in reversing the cognitive deficits of CH pups. The results have unveiled novel roles of TRPC6 in regulating CH‐mediated cognitive deficits.

## 2 Materials and Methods

### 2.1 Animals

Adult female Sprague–Dawley (SD) rats weighing 180 ~ 220 g were provided by the Experimental Animal Center of Nantong University. All animal experiments were approved by the *Animal Care and Use Committee of Nantong University* and the *Animal Care Ethics Committee of Jiangsu Province* (License No. SYXK (Su) f2020–0029). The dams and the pups were housed in standard cages in an air‐conditioned room with a controlled temperature (22°C ± 2°C) on a 12–12 h light–dark cycle and had free access to water and food.

### 2.2 Establishment of CH Model of Rat Pups

Congenital hypothyroidism model of rat pups was prepared by the supply of 0.02% methimazole (MMI, Sigma‐Aldrich, Cat# M8506) in the drinking water of pregnant dams from the 9th day of gestation (E9). The control rats only drank clean water until the 21st day of the postnatal period (P21). Serum of subjects was collected from dams at day 18 of gestation and pups at P1, P7, and P21 to measure the contents of T4 and TSH.

For administration of drugs, the CH pups at P21 were intraperitoneally injected with hyperforin (MCE, Cat# HY‐116330A) at a dose of 2.5 mg/kg once a day for 7 consecutive days, before it was dissolved in the 10% DMSO (Sigma‐Aldrich, Cat# D2650), 40% PEG300 (MCE, Cat# HY‐Y0873), 5% Tween‐80 (MCE, Cat# HY‐Y1891), and 45% saline. The subjects were then tested by behavioral experiments.

### 2.3 Nissl's Staining

The brain of CH rat pups was dissected and quickly put into 4% paraformaldehyde for fixation at 4°C for 24 h. Then, the samples were gradiently dehydrated with alcohol, treated with xylene, and embedded with paraffin. The samples were cut into 4 μm thick slices and deparaffinized by three xylene immersions and gradient alcohol. Then, the sections were washed with ddH_2_O and immersed in tar violet staining reagent for 20 min. Subsequently, they were rinsed with ddH_2_O and separated color with 1% glacial acetic acid. The sections were finally soaked in xylene three times, followed by a seal with neutral glue. The positive signals were observed under the microscope (ZEISS, axio image M2).

### 2.4 Golgi Staining

Golgi staining was performed according to the instructions of the FD Rapid GolgiStain Kit (FD Neuro Technologies, Cat# PK‐401). The tissues of rat pups were immersed in solutions A and B at room temperature for 2 weeks and then immersed in solution C at 4°C for 72 h. The hippocampus was sectioned at 100 μm and pasted on a 3% gelatin slide (Beyotime, Cat# ST1339). The slices were dried at room temperature for 2 days, followed by dyeing with a mixture of solutions D, E, and distilled water (1:1:3) for 10 min. After dehydration with gradient ethanol, neutral gum was used to seal the sections. The hippocampal dendritic spines were observed under a microscope (Leica, DM4B).

### 2.5 Primary Culture of Hippocampal Neurons

To culture hippocampal neurons, E18 pregnant rats were anesthetized with isoflurane, followed by dissection of the fetal rats. The fetal rats were transferred to Dulbecco's Modified Eagle's Medium (DMEM)‐high glucose medium (Sigma‐Aldrich, Cat# D6546), and the brain was dissected out and transferred to Hank's solution. Subsequently, the hippocampus of the fetal rats was isolated on ice under the microscope and placed in 0.125% trypsin for digestion in the incubator at 37°C. The digestion was terminated by the addition of DMEM high glucose medium containing fetal bovine serum (FBS, Gibco, Cat# 16140071) into the solution. The dissociated cells were centrifuged at 1000 rpm for 4 min and were resuspended by the addition of 2 mL medium at 37°C. The cells were filtered and counted before they were inoculated into six‐well or 24‐well plates (1 × 10^4^ cells/cm^2^) coated with poly‐D‐lysine (PLL, Sigma‐Aldrich, Cat# P4707). The medium was discarded after 4 ~ 6 h and was replaced by neurobasal (Gibco, Cat# A3582901) containing B‐27 (Gibco, Cat# A3582801). The primary neurons were cultured at 37°C, 5% CO_2_, and 95% air atmosphere, and the medium was replaced every 2–3 days.

### 2.6 siRNA Transfection and Drug Treatment of Primary Neurons

The siRNA transfection of the primary neurons was performed according to the manufacturer's protocol (RiboBio, Cat# C10511‐05). Cells were cultured in a six‐well plate, and 15 μL of siRNA was added to the 120 μL of riboFECT CP Buffer, followed by the addition of 12 μL riboFECT CP Reagent. After cell incubation for 10 ~ 15 min at room temperature, the transfection complex was added to the B‐27‐containing neurobasal medium to keep the final concentration of siRNA at 150 nM. The neurons were cultured at 37°C in 5% CO_2_ and 95% air. After 24 h, RNA was extracted to determine the transfection efficiency.

For drug treatment, the TRPC6 activator hyperforin (0.3 μM—1 μM, MCE, Cat# HY‐116330A), TR antagonist 1 (5 μM, MCE, Cat# HY‐111443), or T3 (5 nM, MCE, Cat# HY‐A0070A) was added to the neuron culture medium for the desired experiments.

### 2.7 Quantitative Polymerase Chain Reaction (Q‐PCR)

Total RNA was extracted from the hippocampus or cultured neurons with Trizol (Sigma‐Aldrich, Cat# T9424). The RNAs were reverse‐transcribed into cDNA in a 20 μL reaction system using the reverse transcription kit (Vazyme, Cat# R423‐01) according to the instructions. The cDNA was diluted 1:3 before Q‐PCR detection. Sequence‐specific primers were designed and synthesized by Generay (Shanghai, China). The details were shown as follows: for *trpc6*, forward primer 5‐CCC CAA TCA TTC TGG CTG CT‐3′, reverse primer 5‐GAG TCT CTG CAT CCC CGT TC‐3′; for *gapdh*, forward primer 5‐ACA GCA ACA GGG TGG TGG AC‐3′, reverse primer 5‐TTT GAG GGT GCA GCG AAC TT‐3′; for *camkIV*, forward primer 5‐TGG AGG CAG TTG CTT ACC TG‐3′, reverse primer 5‐CCT CGG AGA ATC TCA GGT GC‐3′; for *creb*, forward primer 5‐GCA GGT GAC TGA GGA GCT TGT‐3′, reverse primer 5‐ACC TGT GGC TAA T‐3′; for *egr3*, forward primer 5‐CTC AGA TGG CTA CAG AGA ATG TG‐3′, reverse primer 5‐ACC AGT TGG AAG GAG AGT CG‐3′; for *bdnf*, forward primer 5‐GTC CCG GTA TCA AAA GGC CA‐3′, reverse primer 5‐ATC CTT ATG AAC CGC CAG CC‐3′. The 2 × ChamQ Universal SYBR qPCR Master Mix (Vazyme, Cat# Q711‐02) was used for the Q‐PCR assay in the Light Cycler 96 real‐time PCR system (Roche, LightCycler 96). Reactions were performed with one initial denaturation cycle at 94°C for 5 min, followed by 40 cycles of 94°C for 30 s each cycle, 60°C for 30 s, and 72°C for 30 s. After the amplification reaction was completed, the CT value and amplification curve were automatically analyzed by the system, and the expression levels were normalized using endogenous *gapdh* according to the 2^−ΔΔt^ algorithm.

### 2.8 Western Blot

Proteins were extracted from the cells with RIPA lysis buffer (MCE, Cat# HY‐K1001) containing PMSF (MCE, Cat# HY‐B0496). The primary neurons were scraped with a cytoskeletal scraper on ice, vortexed for 30 min at 4°C, and centrifuged at 12000 rpm for 15 min. Protein concentration was determined and quantified according to the BCA method (Beyotime, Cat# P0006). The samples were then heated at 95°C for 5 min and separated by electrophoresis on a 10% SDS‐PAGE gel (Beyotime, Cat# P0012AC), followed by a transfer to a PVDF membrane (Millipore, Cat# IPVH00010). The membrane was blocked with 5% skimmed milk (Beyotime, Cat# P0216) for 1 h and incubated in primary antibody at 4°C for 24 h. Primary antibodies were used as follows: rabbit‐anti‐CaMKIV (1:1000, Proteintech, Cat# 13263–1‐AP), mouse‐anti‐EGR3 (1:500, Santa Cruz, Cat# sc‐390967), rabbit‐anti‐BDNF (1:1000, Abcam, Cat# ab108319), mouse‐anti‐GAPDH (1:5000, Proteintech, Cat# 60004–1‐Ig). After 3 washes with TBST for 10 min each, the PVDF membrane was incubated with secondary antibody goat‐anti‐mouse HRP or goat‐anti‐rabbit HRP (1:1000, Beyotime, Cat# A0216, Cat# A0208) for 2 h at room temperature. After the PVDF membrane was washed, the Western blot bands were detected using the ECL kit (Beyotime, Cat# P0018). The protein levels were normalized to an endogenous GAPDH.

### 2.9 Subcellular Fractionation

Extraction of nuclear and cytosolic protein from the cells was carried out according to the procedure of the Nuclear and Cytoplasmic Protein Extraction Kit (Beyotime, Cat# P0028). Briefly, the cells were washed with ice‐cold PBS, followed by lysis in 200 μL cytoplasmic protein extraction agent A, supplemented with 1 mM PMSF on ice for 15 min before vortexing for 5 s. Then, the cytoplasmic protein extraction agent B was added, and the mixture was vortexed for 5 s prior to incubation on ice for 1 min. Samples were centrifuged at 12000 g at 4°C for 5 min, and the supernatant (cytosolic fraction) was immediately collected. The nuclear fraction was resuspended in nuclear protein extraction agent. After several vortexing times vortex for 30 min, centrifugation was performed at 12000 g for 10 min. The supernatants containing the nuclear extracts were thus collected. Proteins of nuclear and cytosolic extracts were determined by Western blot. Primary antibodies were used as follows: rabbit‐anti‐CREB (1:1000, Proteintech, Cat# 12208–1‐AP), mouse‐anti‐GAPDH (1:5000, Proteintech, Cat# 60004–1‐Ig), rabbit‐anti‐Lamin B1 (1:1000, Proteintech, Cat# 12987–1‐AP). The GAPDH or Lamin B1 was used as the internal control of cytoplasmic or nuclear proteins, respectively.

### 2.10 Immunofluorescence

Brain tissue of the subjects was dissected and fixed in 4% paraformaldehyde solution at 4°C for 24 h. Then, they were dehydrated in 10%, 20%, and 30% sucrose solution. After being embedded with Tissue‐Tek Optimal Cutting Temperature compound (OCT, Sakura, 4583), the tissues were sectioned at 12 μm by a cryostat (Leica, CM3050S). The sections were dried in an oven at 37°C. As for immunofluorescence staining of primary neurons, the cells were grown on small round glass slides. The slides were washed with PBS three times, each for 5 min. Solution containing 3% BSA (BioFroxx, Cat# 4240GR100), 0.1% Triton X‐100 (Beyotime, Cat# ST797), and 10% normal goat serum (Bioss, Cat# C‐0005) in 0.01 M PBS was added to the tissue for 1 h at 37°C or 24 h at 4° *C. Triton* X‐100 was absent from immunofluorescence of membrane proteins. The primary antibodies were used as follows: mouse anti‐MAP2 (1:500, Sigma‐Aldrich, Cat# M9942), rabbit anti‐TRPC6 (1:100, Proteintech, Cat# 18236–1‐AP), or mouse anti‐NeuN (1:500, Abcam, Cat# ab104224), and rabbit anti‐p‐CREB (1:500, CST, Cat# 9198). After 3 washes of the slices with PBS for 10 min each, the secondary antibody was incubated at 4°C for 16 h before being sealed with mounting medium containing DAPI (Abcam, Cat# ab104139). The secondary antibodies were used as follows: Alexa Fluor 488‐labeled donkey anti‐rabbit IgG (1:400, Abcam, Cat# ab150073) or Cy3‐labeled goat anti‐mouse IgG (1:400, Abcam, Cat# ab97035). Immunostaining was observed under the microscope (Zeiss, Axio image M2) or the laser confocal microscope (Zeiss, LSM 900).

### 2.11 Calcium Assay

The experiment was performed according to the procedure of Fluo‐8 Calcium Flux Assay Kit—No Wash (Abcam, Cat# ab112129). All kit components were thawed at room temperature before use, and the Fluo‐8 stock solution was prepared. The primary hippocampal neurons at 1.2 × 10^5^ cells were inoculated in Petri dishes (diameter at 2 cm, Biosharp, Cat# BS‐20‐GJM), followed by incubation with Fluo‐8 stock solution for 30 min at 37°C, and another 30 min at room temperature according to the manufacturer's instructions. Then the medium was changed to calcium‐free D‐Hanks' balanced salt solution (HBSS, Biosharp, Cat# BL559A) before transferring the dishes to a live cell workstation. The laser confocal microscope (Zeiss, LSM 900) was used to catch calcium imaging with fluorescence intensity at 488 nm. A total of 4 fields were chosen for each shooting, and one image was taken every 8 s for each field. The fluorescence intensity within 232 s was recorded. Relative changes in fluorescence intensity were calculated and normalized against the control.

### 2.12 Bioinformatics Analysis

Gene network was created using the STRING website (https://string‐db.org/) and Cytoscape (Version 3.10.0) software, based on differentially expressed genes related to the biological processes including anatomical structure morphogenesis, negative regulation of growth, developmental processes, and response to hormones. The Cytoscape plugin Cytohubba was used to identify the hub factor through the Cytoscape plugin IRegulon.

### 2.13 Behavioral Tests

For Morris water maze tests (MWM), the CH subjects at 24 weeks were trained for 4 days each four times after the beginning of the tests. In the place navigation test, the rats were put into the water with their backs to the platform at an entry point. Then, they were observed, and recorded the time and distance needed to find and climb onto the platform (escape latency and distance) were recorded. If the rats failed to find the platform within 90 s, they were guided to the platform, and the escape latency was recorded as 90 s. The escape latencies of the rats were recorded on days 1–4. In the spatial probe test, the platform was removed on day 5, and the times of crossing the original platform location in the pool, duration, and latency within 90 s were recorded. Data collection and processing were performed using a MWM image automatic monitoring system.

For the Novel object recognition (NOR) test, the subjects were put into the test room for environmental adaptation at 24 h before tests. To avoid the influence of odor or potential stress, the environment was cleaned and kept quiet and tidy. Two squared wooden blocks with the same volume were put in a box, and the subjects were moved in and performed to the Noldus object recognition experiment. The rat pups were allowed to explore the objects freely in the box for 10 min. After the rats rested for 1 h, one of the wooden blocks was replaced with a new cylindrical one. The subjects were allowed to explore the objects for another 10 min, and the time for the new and the old objects was recorded. MWM and NOR were analyzed using the Ethovision XT 16 system (Noldus Information Technology, Netherlands).

### 2.14 Statistical Analysis

Statistical analysis was performed using GraphPad Prism 8 software (San Diego, CA, USA). All data were presented as mean ± standard error of mean (M ± SEM). Comparisons between two groups following normal distribution were analyzed by a two‐tailed unpaired Student's t test or the Mann–Whitney test when the distribution was not parametric. Differences between multiple groups were analyzed by one‐way analysis of variance (ANOVA) or two‐way ANOVA, followed by Tukey's or Dunnett's post hoc test. The Kruskal–Wallis test was used when the distribution was not parametric. P value < 0.05 was considered statistically significant (*p < 0.05).

## 3 Results

### 3.1 CH Of Rat Pups Results in Deficient Expression of TRPC6 in the Dentate Gyrus (DG) Neurons of Hippocampus

**FIGURE 1:**
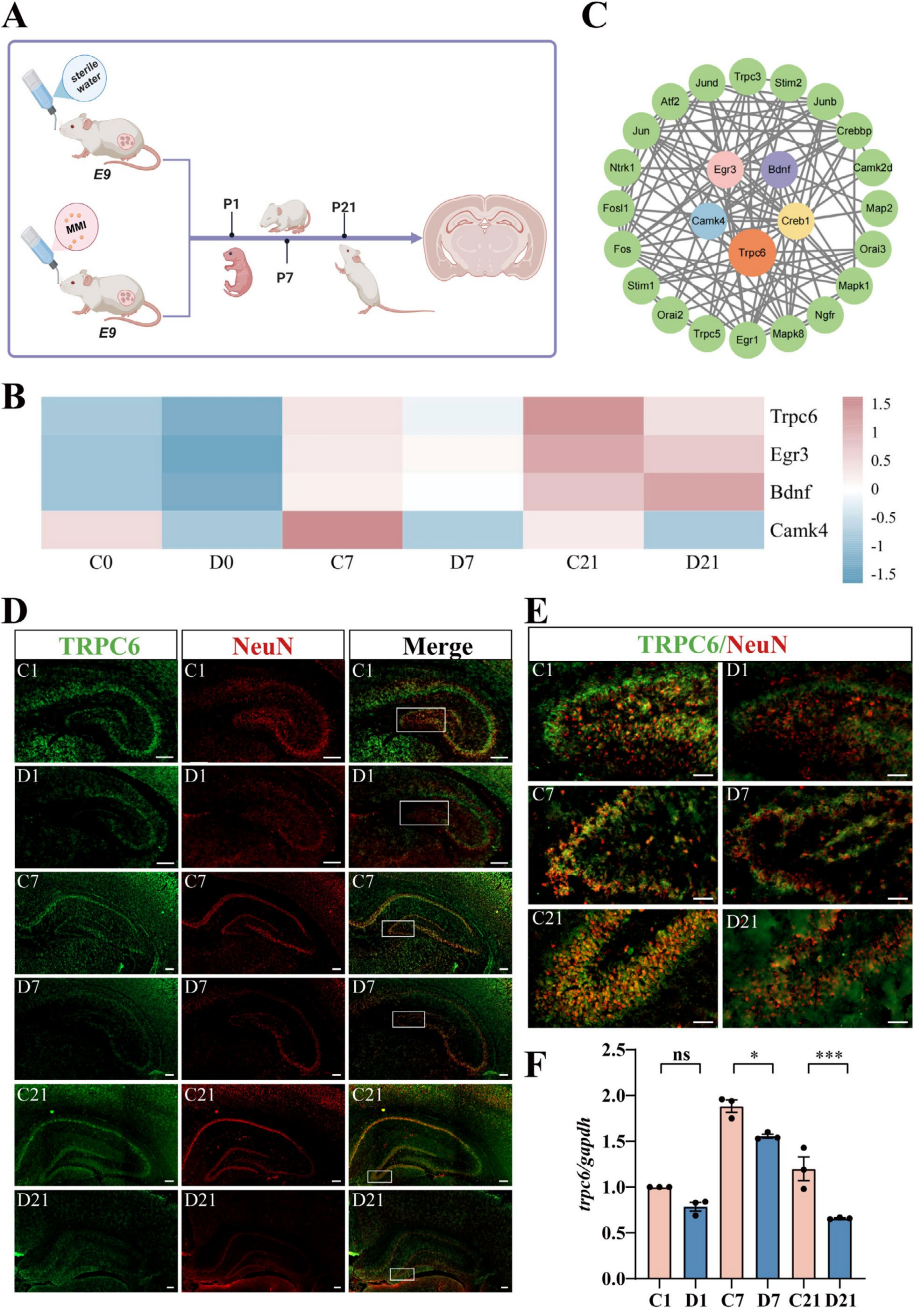
Analysis of TRPC6 expression in the hippocampal dentate gyrus of CH pups. (A) Schematic diagram of inducing CH rat pups. (B) Gene network of DEGs in hippocampal tissues of CH rat pups at P1, P7 and P21. (C) Heatmap of *trpc6*, *camkIV*, *creb* and *bdnf* selected from DEGs in the hippocampus tissues of P1, P7, and P21 rat pups following CH. (D, E) Co‐localization of TRPC6 with NeuN^+^ neurons in the hippocampus of CH rats at different stages by immunostaining. C1/7/21 indicates control, and D1/7/21 indicates CH. The rectangles in (D) indicate the magnification in (E). Scale bars: 200 μm in (D); 50 μm in (E). (F) The transcriptional levels of TRPC6 in the hippocampus of P1/7/21 rat pups by RT‐PCR. Quantities were normalized to endogenous *gapdh*. Experiments were performed in triplicates. Data are expressed as mean ± SEM, *p < 0.05, **p < 0.01, and ***p < 0.001, two‐way analysis of variance followed by Tukey's *post hoc* test.

To gain an insight into the relationship between CH‐mediated dysmorphogenesis of the hippocampus and TRPC6 deficiency, CH rat pups were successfully induced by adding 0.02% methimazole (MMI) to the drinking water of dams from gestation day 9 (E9) to postnatal day 21 (P21), as was shown in the previous works (Figure 1A) [22, 23]. Determination of serum thyroid‐stimulating hormone (TSH) and total thyroxine (T4) from both dams and pups confirmed the consistency with the clinicopathologic indicators of hypothyroidism [22]. Hippocampal tissues of CH pups at P1, P7, and P21 were collected for transcriptome sequencing, and a total of 38 (P1), 147 (P7), and 385 (P21) differentially expressed genes (DEGs) were identified at the three time points [22]. A gene network using DEGs involved in anatomical structure morphogenesis, negative regulation of growth, and response to hormone was constructed by STRING database. It was seen that TRPC6 was highlighted as a core molecule by interacting with CaMKIV, CREB, and BDNF (Figure 1B,C) indicating that TRPC6 possibly mediates abnormal hippocampal development of CH pups through regulating the CaMKIV/CREB/BDNF axis.

Several studies display that TRPC6 is abundantly expressed in the hippocampal neurons to act roles in development [36]. To examine whether CH of the pups led to aberrant expression of TRPC6 in the hippocampus, immunofluorescence was used to detect the TRPC6 abundance within NeuN‐positive neurons. Results revealed that TRPC6 was ubiquitously distributed in the hippocampal CA1 and CA3, as well as DG regions at P1, P7, and P21 rat pups (Figure 1D). However, its abundance within NeuN‐positive granule neurons of the DG region was significantly reduced by the CH (Figure 1E). The transcriptional level results also validated the immunohistochemical findings (Figure 1F). The data indicate that CH downregulates TRPC6 expression in DG neurons in association with impaired hippocampal development.

### 3.2 TRPC6 Deficiency Decreases Dendritic Spine Growth of Rat Hippocampal Granule Neurons

**FIGURE 2:**
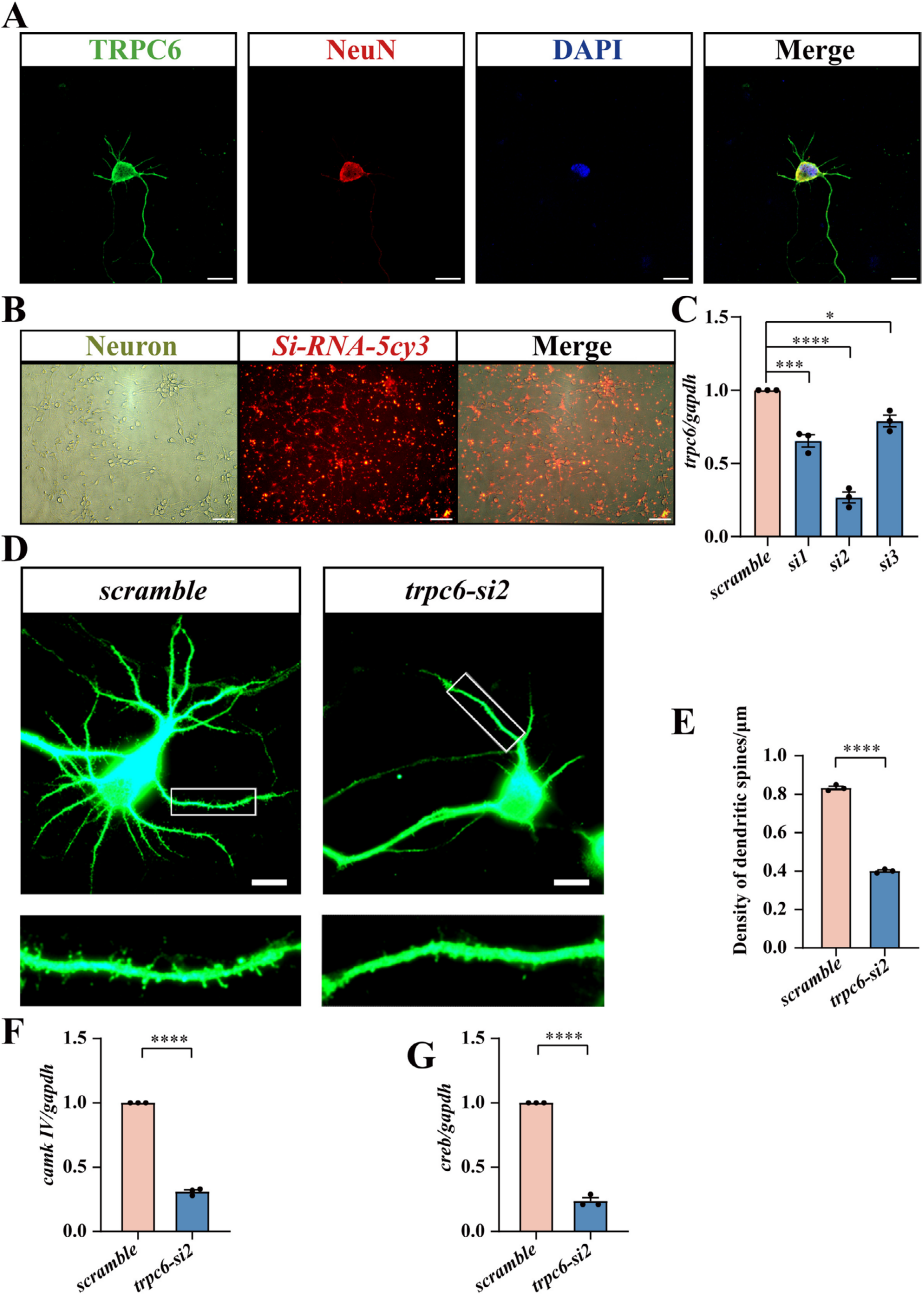
Knockdown of TRPC6 affects dendritic spine growth of hippocampal neurons. (A) Immunostaining of TRPC6 in primary hippocampal neurons. (B) Detection of siRNA transfection efficiency by using 150 nM 5‐Cy3 oligonucleotide for 24 h. (C) Analysis of interference efficiency of three TRPC6 siRNA oligonucleotides using RT‐PCR, and the siRNA2 (*trpc6‐si2*) was chosen for the subsequent experiments. Quantities were normalized to endogenous *gapdh*. Data are expressed as mean ± SEM, *n* = 3, *p < 0.05, **p < 0.01, ***p < 0.001, and ****p < 0.0001, oneway analysis of variance followed by Dunnett's *post hoc* test. (D) Immunostaining of primary hippocampal granule neurons with MAP2 to detect dendritic spine density following *trpc6‐si2* knockdown for 24 h. The rectangle indicates region magnified. (E) Statistical analysis of (D). Spine density was analyzed in triplicates each 15 fields. Scale bar: 10 μm in (A) and (D); 100 μm in (B). (F, G) Transcriptional analysis of *camkIV* and *creb* in primary hippocampal granule neurons after *trpc6* knockdown for 24 h. Quantities were normalized to endogenous *gapdh*. Six pups of each group were used to isolate hippocampal neurons. Data are expressed as mean ± SEM, *n* = 3, *p < 0.05, **p < 0.01, ***p < 0.001, and ****p < 0.0001, two‐tailed unpaired Student's *t*‐test.

To elucidate the exact roles of TRPC6 in the growth of hippocampal neurons, the hippocampal granule neurons from fetal rats were cultured and interfered with TRPC6 siRNAs. The primary granule neuron was shown to express TRPC6, as was examined by immunofluorescence (Figure 2A). Transfection efficiency determined by siRNA‐5cy3 reached over 81.2% (Figure 2B), and the knockdown efficiency of TRPC6 in the neurons reached more than 70% by one (*trpc6‐si2*) out of three siRNAs (Figure 2C). Results demonstrated that knockdown of TRPC6 expression in granule neurons for 24 h significantly reduced the density of dendritic spines, which were labeled by MAP2 antibody (Figure 2D,E). Accordingly, the expression of *CaMKIV* and *CREB* was remarkably decreased in comparison with the scramble (Figure 2F,G). The data indicate that TRPC6 deficiency in hippocampal neurons affects the growth of dendritic spines by regulation of the CaMKIV/CREB signaling pathway.

### 3.3 Activation of TRPC6 Increases Dendritic Spine Density of Hippocampal Neurons

**FIGURE 3:**
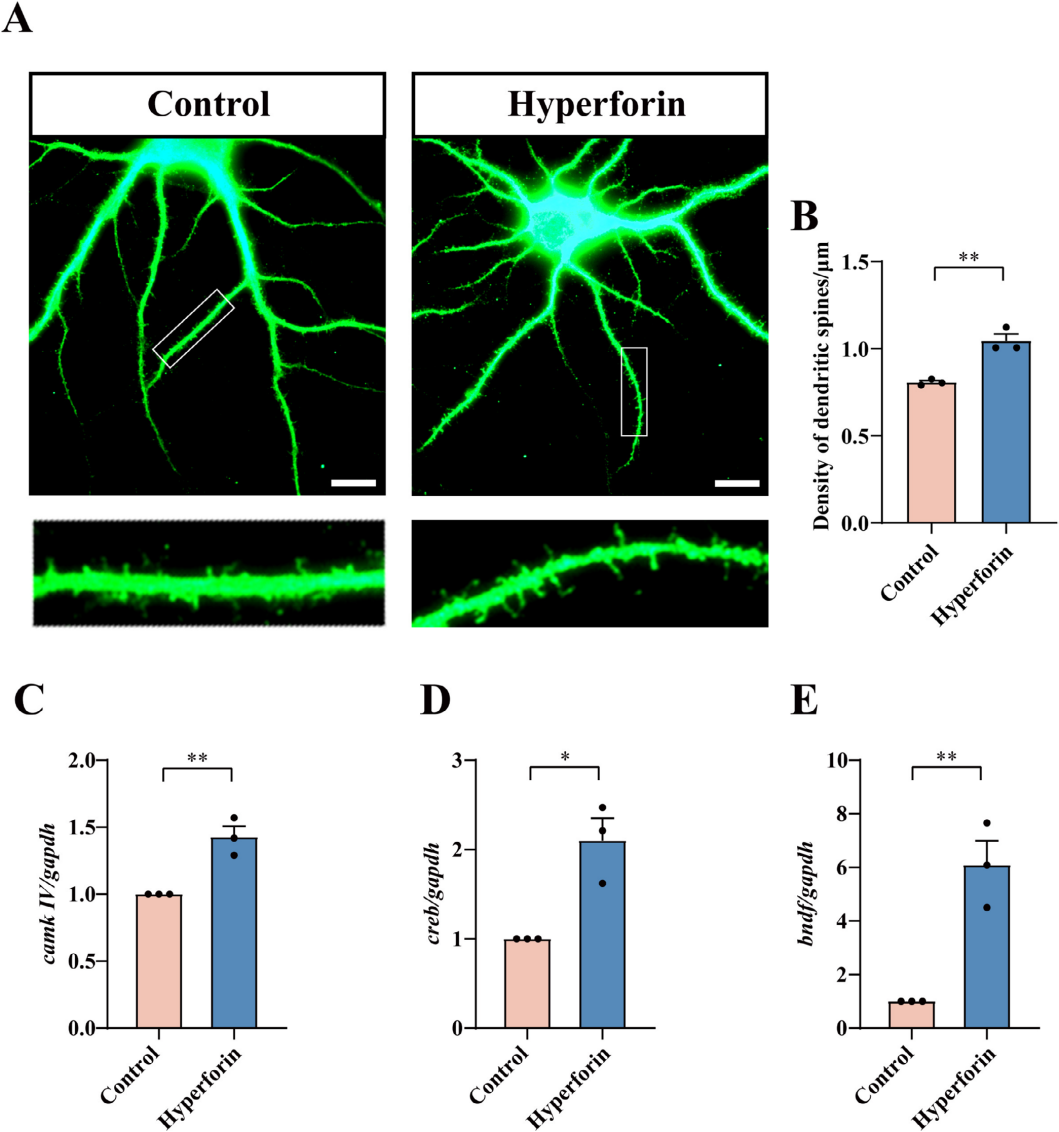
Effect of TRPC6 activator on the dendritic spine density of hippocampal DGCs. (A, B) Primary DGCs were cultured for 5 d, followed by incubation with 0.3 μM TRPC6 activator hyperforin (Hyp) for 24 h. The dendritic spine density was observed by immunofluorescence with MAP2 antibody. The rectangle indicates region magnified. Spine density was analyzed in triplicates each 15 fields. (C‐E) Transcriptional analysis of *camkIV*, *creb* and *bdnf* after treatment of the neurons with 1 μM hyperforin for 1 h. Quantities were normalized to endogenous *gapdh*. Scale bar: 10 μm in (A). Experiments were performed in triplicates. Data are expressed as mean ± SEM, *p < 0.05 and **p < 0.01, two‐tailed unpaired Student's *t*‐test.

To further address the effects of TRPC6 activation on dendritic spine growth of hippocampal neurons, the TRPC6 agonist hyperforin (Hyp) was applied to stimulate primary granule neurons. Hyperforin has been shown to specifically activate TRPC6 and acts protective roles on the neurons [37, 38, 39]. Exposure of the hippocampal neurons to 0.3 μM hyperforin for 24 h revealed that activation of TRPC6 was able to increase the density of dendritic spines and facilitate the morphological changes from thin and filopodia‐like to mature stubby and mushroom dendritic spines (Figure 3A,B). As was expected, activation of TRPC6 markedly promoted the upregulation of *CaMKIV* and *CREB* expression, as well as downstream *BDNF* (Figure 3C–E). The data indicate that TRPC6 contributes to the dendritic spine growth of hippocampal neurons through activation of CaMKIV signaling.

### 3.4 Hyperforin Was Able to Rescue the Abnormal Dendritic Spine Growth of Hippocampal Granule Neurons from CH Rats

**FIGURE 4:**
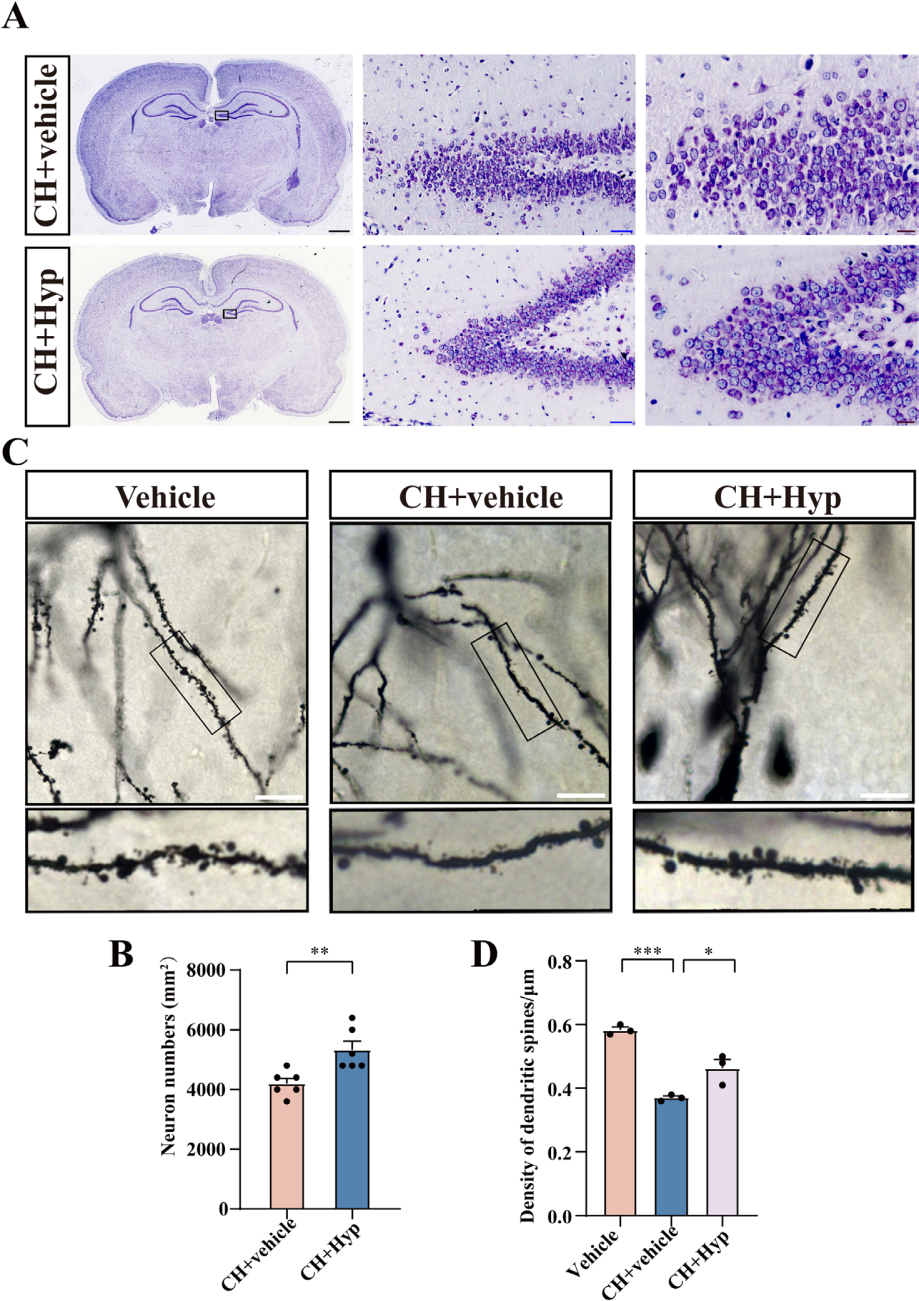
Effects of TRPC6 agonist on the number of neurons in DG and the growth of dendritic spines in CH pups. CH pups at 21 d were injected intraperitoneally with 2.5 mg/kg hyperforin for 7 d. (A) The morphology and number of neurons in the DG of the subjects were observed by Nissl's staining. The rectangle indicates the DG region magnified. Scale bars: 1000 μm; 50 μm or 20 μm in magnification. (B) Statistical analysis of (A), 15 fields in each section were subjected to the statistical analysis of neuron number. Data are expressed as mean ± SEM, *n* = 6, **p < 0.01, two‐tailed unpaired Student's *t*‐test. (C) Detection of the dendritic spine density of DGCs in the DG of the subjects following injection of hyperforin by Golgi staining. The rectangle indicates region magnified. Scale bar: 25 μm in (C). (D) Statistical analysis of (C). Spine density was analyzed in triplicates each 15 fields. Data are expressed as mean ± SEM, *n* = 3, *p < 0.05, **p < 0.01, and ***p < 0.001, oneway analysis of variance followed by Dunnett's *post hoc* test.

To understand whether the TRPC6 agonist is able to protect the hippocampal granule neurons from CH‐mediated impairment of dendritic spine growth, the CH rat pups at P21 were intraperitoneally injected with 2.5 mg/kg hyperforin (Hyp). The control was injected with equivalent vehicle containing 10% DMSO, 40% PEG300, 5% Tween‐80, and 45% saline. Results of Nissl's staining at 7 days demonstrated that hyperforin could increase the number of DG neurons in CH pups (Figure 4A,B). In the meantime, Golgi staining revealed that the dendritic spine density of granule neurons was significantly increased by the hyperforin treatment (Figure 4C,D). It was notable that the morphology of the spines was more round and uniform than that of the control (Figure 4C). The data indicate that activation of TRPC6 is able to rescue the abnormal dendritic spine growth of hippocampal granule neurons in CH pups.

### 3.5 TRPC6 Regulates Neuronal CaMKIV/CREB Signaling Pathway Through Modulating Ca^2+^ Inward Flow

**FIGURE 5:**
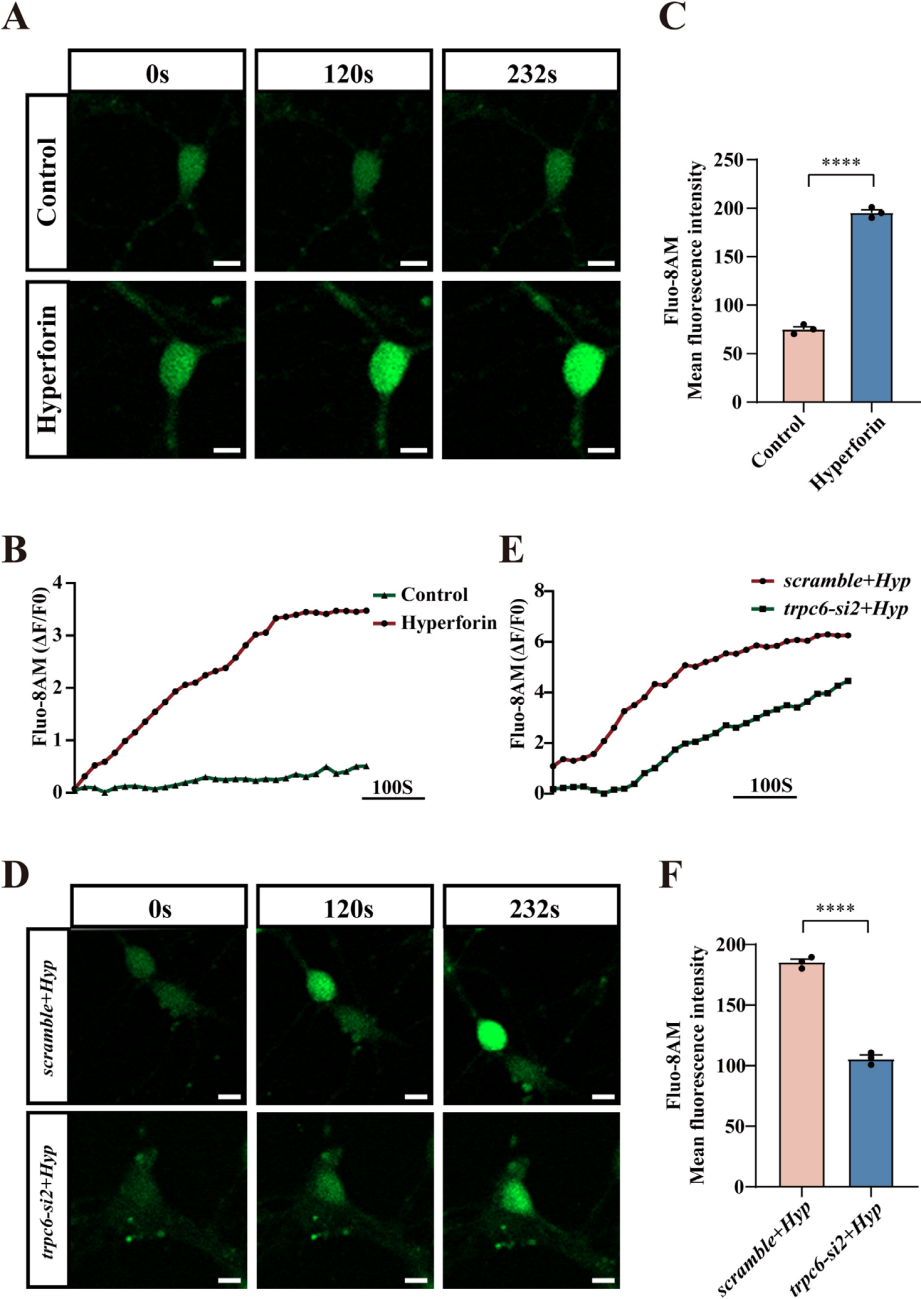
Effect of TRPC6 activation on calcium transients in hippocampal neurons of rat pups. (A) Calcium transients in primary DGCs were stimulated with 10 μM hyperforin, and the cells were stained with Fluo‐8 AM (green) to indicate intracellular Ca^2+^ level. Scale bar: 10 μm in (A). (B) The fluorescence intensity changes in (A). (C) Analysis of fluorescence intensity using neuronal calcium imaging within 232 s. (D) Detection of neuronal calcium transients following knockdown of *trpc6* for 24 h and then treatment with hyperforin. Scale bar: 10 μm in (D). (E) The fluorescence intensity changes in (D). (F) Analysis of fluorescence intensity using neuronal calcium imaging following knockdown of *trpc6* for 24 h and then treatment with hyperforin. The fluorescence intensity was quantified from 10 cells. Time‐lapse was collected every 8 s for a total of 232 s. Experiments were performed in triplicates. Data are expressed as mean ± SEM, *p < 0.05, **p < 0.01, ***p < 0.001, and ****p < 0.0001, two‐tailed unpaired Student's *t*‐test.

As a cation channel, TRPC6 is highly permeable to Ca^2+^. To unveil the mechanism of TRPC6 in regulating the growth of dendritic spines of hippocampal granule neurons, 10 μM hyperforin was used to stimulate these primary cells. The agonist at this concentration has been shown to be sufficient in inducing intracellular calcium transients in cortical neurons [40, 41]. By using the Fluo‐8 AM novel calcium fluorescent probe, the calcium imaging was monitored at 8 s intervals. The results demonstrated that exposure of the cells to hyperforin resulted in a rapid calcium transient response and a significant increase in fluorescence intensity in the rat hippocampal neurons (Figure 5A–C). Whereas knockdown of TRPC6 for 24 h, followed by incubating the cells with 10 μM hyperforin, markedly attenuated neuronal calcium inward flow (Figure 5D–F). Although the intracellular calcium transients were still present, they might be attributed to the Store‐Operated Calcium Entry (SOCE) effect of the endoplasmic reticulum calcium pool [42, 43]. The data indicate that activation of TRPC6 is able to induce the calcium influx of hippocampal neurons.

**FIGURE 6:**
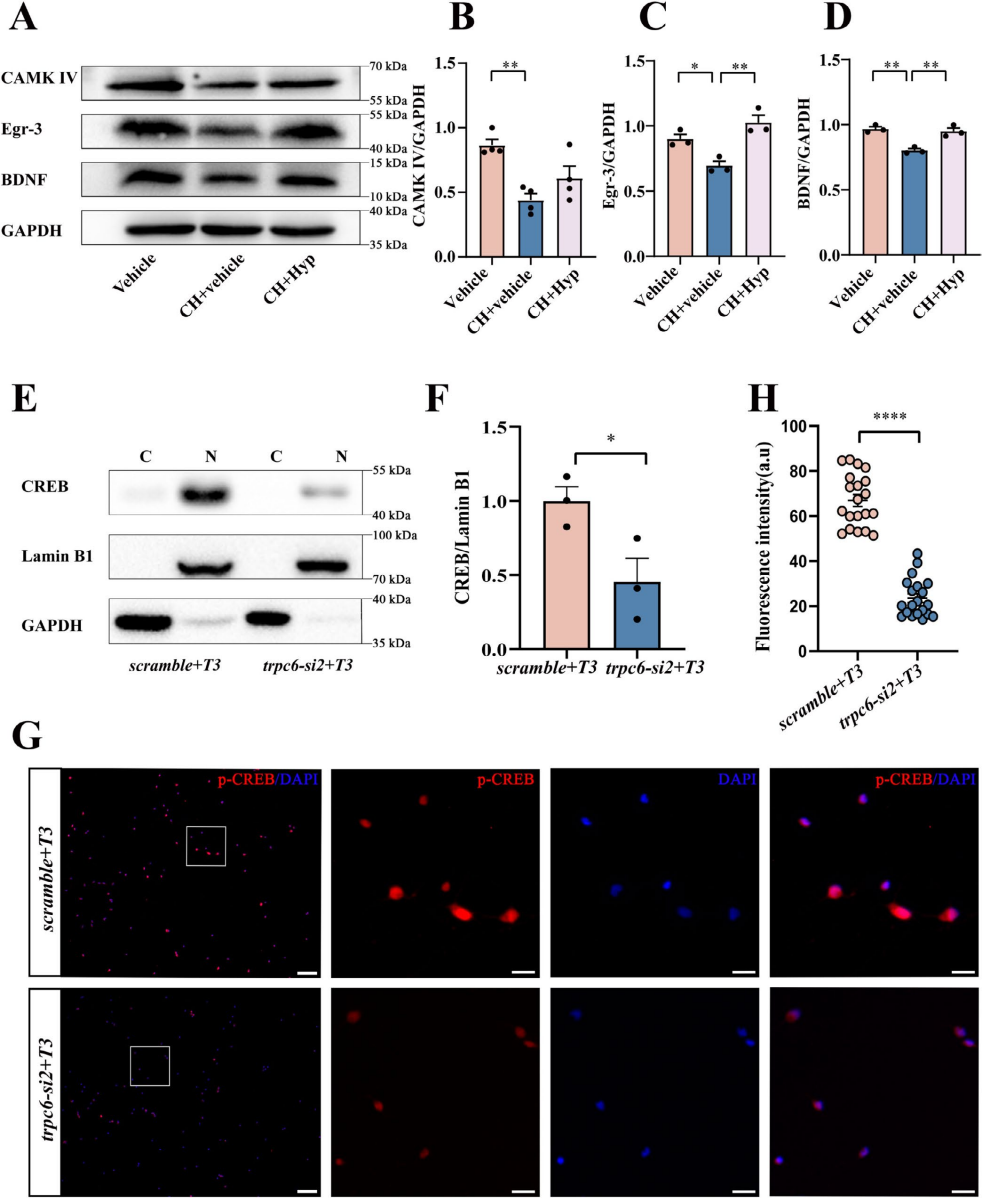
TRPC6 modulates the CaMKIV/CREB pathway in hippocampal tissue of CH pups and neurons. (A) Western blot analysis of CaMKIV, Egr‐3 and BDNF protein levels at hippocampal tissues from CH pups. CH pups at 21 d were injected intraperitoneally with 2.5 mg/kg hyperforin for 7 d. (B‐D) The quantitative analysis of (A). Quantities were normalized to endogenous GAPDH. Data are expressed as mean ± SEM, *n* = 3, *p < 0.05, **p < 0.01, oneway analysis of variance followed by Tukey's *post hoc* test. (E) Western blot analysis of CREB in cytosolic and nuclear fraction of primary hippocampal neurons after transfection with *trpc6‐si2* for 24 h, followed by stimulation of 5 nM T3 for 1 h. Quantities were normalized to endogenous GAPDH (cytoplasm) or Lamin B1 (nucleus). (F) The quantitative analysis of (E). Data are expressed as mean ± SEM, *n* = 3, *p < 0.05, two‐tailed unpaired Student's *t*‐test. (G) Immunostaining of p‐CREB following the primary DGCs knockdown of trpc6 for 24 h and then treatment with 5 nM T3 for 1 h. The rectangle indicates region magnified. Scale bar: 100 μm, and 20 μm in magnification. (H) The analysis of fluorescence intensity in (G), *n* = 20, ****p < 0.0001, Mann‐Qhitney test.

Neuronal calcium influx is essential for activation of calcium‐dependent protein kinases (CaMKs) responsible for multiple intracellular signaling cascades. Our previous works revealed that dysregulation of CaMKIV in the CH pups mediated dendritic spine growth impairment of hippocampal neurons [22]. To examine whether hippocampal neuronal CaMKIV/CREB is governed by TRPC6, the CH pups at P21 were intraperitoneally injected with 2.5 mg/kg hyperforin for 7 days, and the hippocampal tissues were collected for Western blot assay. The results showed that protein levels of CaMKIV, Egr‐3, and BDNF were significantly increased by the TRPC6 agonist (Figure 6A–D). To elucidate the direct influence of TRPC6 on the T3‐regulated CaMKIV/CREB axis, the primary granule neurons were transfected with *trpc6‐si2* for 24 h, followed by stimulation of 5 nM T3 for 1 h. Immunoblot analysis of cytosolic and nuclear fractions revealed that *trpc6* silencing abolished T3‐induced upregulation of CREB (Figure 6E,F). In addition, the immunostaining demonstrated that suppression of TRPC6 expression inhibited the accumulation of CREB in the neuronal nucleus (Figure 6G,H). The data indicate that TRPC6 regulates the neuronal CaMKIV/CREB signal axis through inducing Ca^2+^ influx.

### 3.6 T3/TRs Signal Axis Contributes to Regulating TRPC6 Expression in Hippocampal Neurons

**FIGURE 7:**
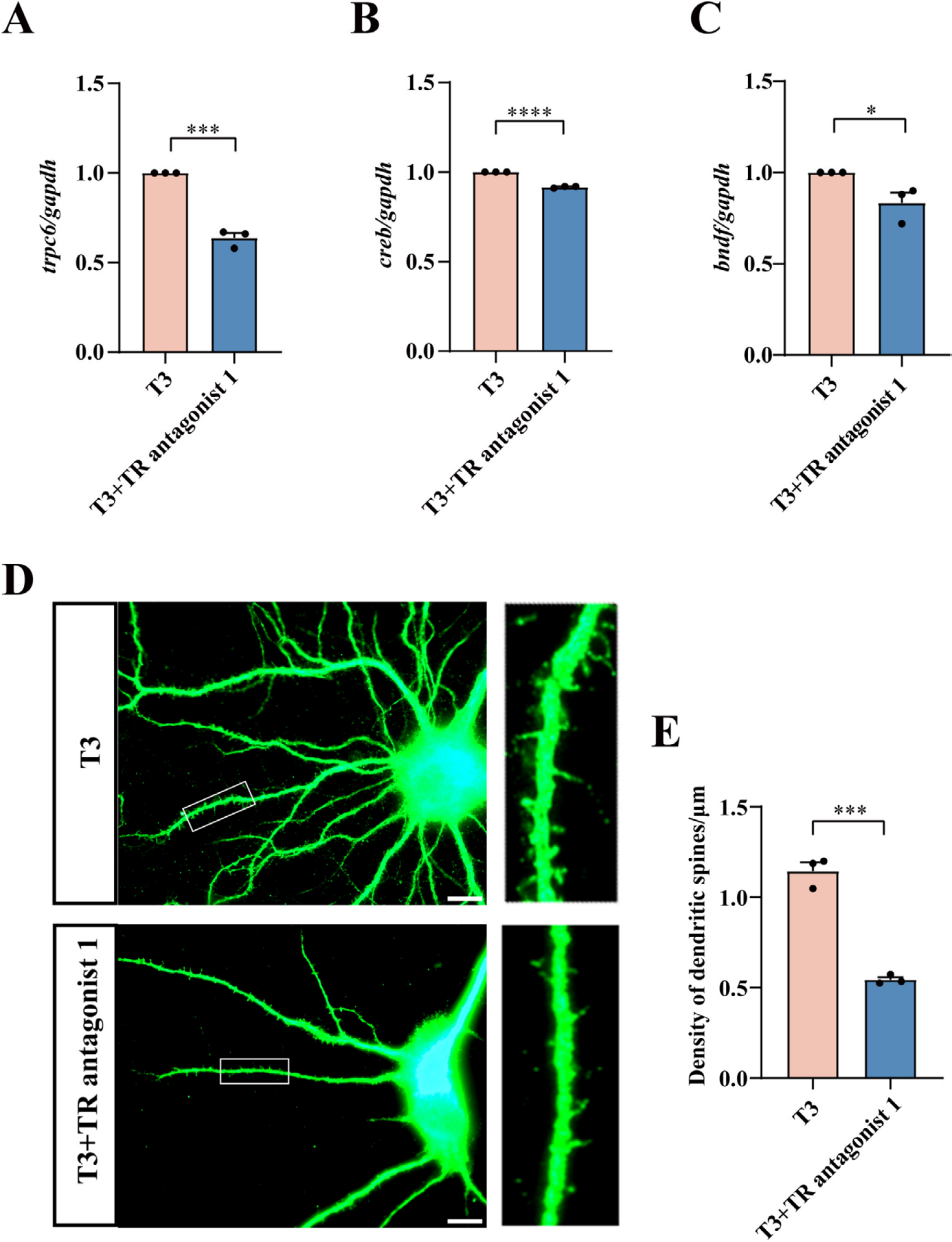
Effects of TRs inhibitors on the expression of TRPC6 and dendritic spine growth in hippocampal neurons. (A‐C) Primary hippocampal neurons were treated with T3 (5 nM) for 1 h, followed by addition of TR antagonist 1 (5 μM) for 12 h, RT‐PCR analysis of *trpc6*, *creb* and *bdnf*. Quantities were normalized to endogenous *gapdh*. (D, E) Morphological and density changes of dendritic spines after treatment of hippocampal neurons with TR antagonist 1 (5 μM) for 12 h. The rectangle indicates region magnified. Scale bar: 10 μm in (D). Data are expressed as mean ± SEM, *n* = 3, *p < 0.05, **p < 0.01, ***p < 0.001, and ****p < 0.0001, two‐tailed unpaired Student's *t*‐test.

So far, the mechanism of TRPC channels in response to T3 stimulation remains elusive. T3 performs physiological functions through interaction with the nuclear receptors TRα and TRβ. To ascertain the potential mechanism of T3‐mediated TRPC6 expression on the hippocampal neurons, the primary cells were stimulated with 5 nM of T3 for 1 h, followed by treatment with 5 μM of TR antagonist 1 (for both TRα and TRβ) for 12 h [44, 45]. The results demonstrated that the addition of TR antagonist 1 significantly attenuated T3‐mediated expression of TRPC6. Accordingly, the expression of downstream CREB and BDNF was also decreased (Figure 7A–C). Immunostaining of MAP2 showed that TR antagonist 1 was able to reduce the density of hippocampal neuronal dendritic spines (Figure 7D,E). The data indicate that T3 mediates the maturation of rat hippocampal neurons by regulating TRPC6.

### 3.7 TRPC6 Agonist Is Efficient in Improving Cognitive Impairment in CH Rats

**FIGURE 8:**
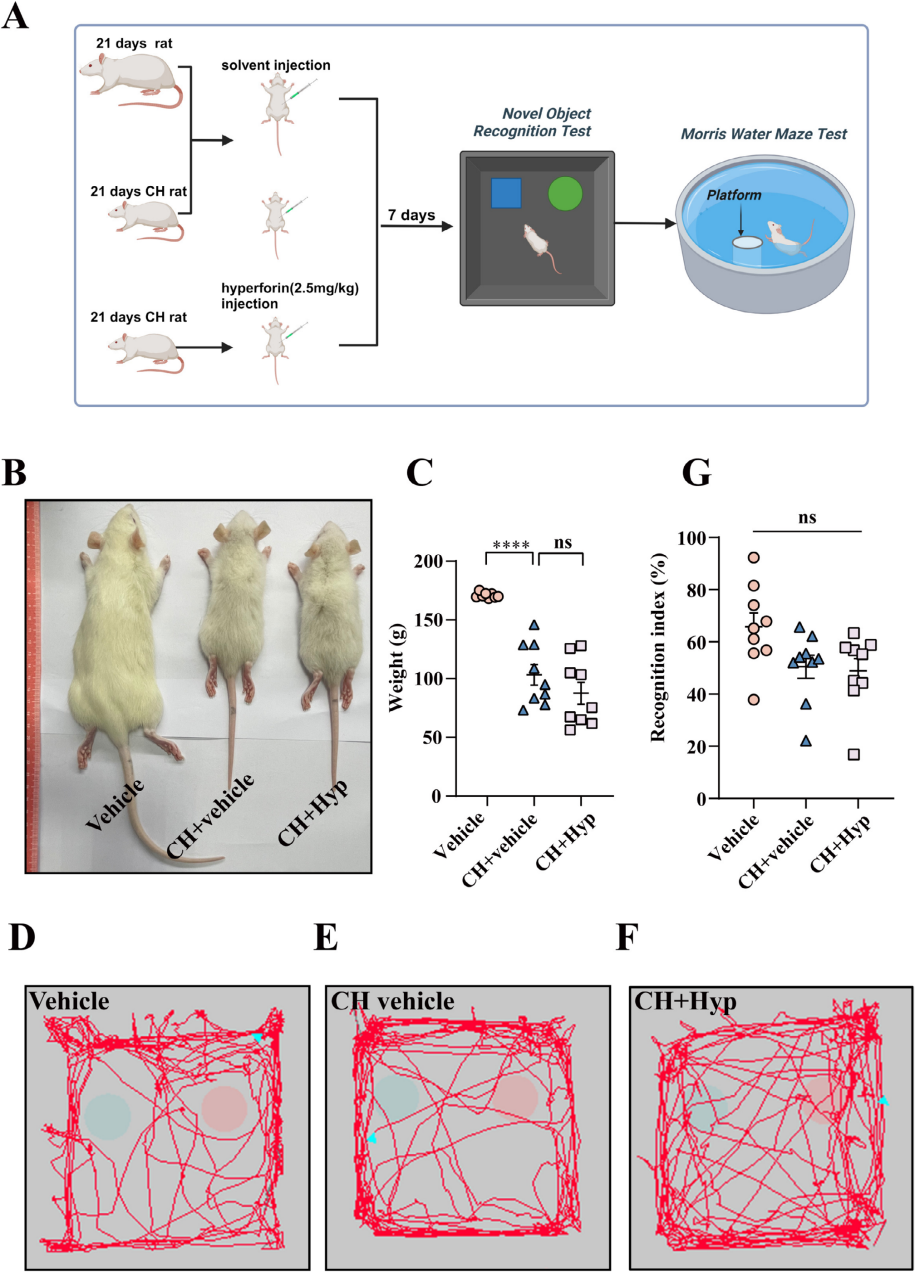
Effects of TRPC6 agonist hyperforin on body weight and NOR of CH pups. (A) Schematic diagram of behavioral test for the CH rat pups. CH pups at 21 d were intraperitoneally injected with hyperforin (2.5 mg/kg), and behavioral tests were performed at 7 d later. (B, C) Body weight of CH pups with or without treatment of TRPC6 agonist. (D‐F) Locomotor trajectories of pups in the NOR test. (G) Recognition index of pups in the NOR test. Data are expressed as mean ± SEM, *n* = 9, *p < 0.05, **p < 0.01, ***p < 0.001, and ****p < 0.0001, oneway analysis of variance followed by Tukey's *post hoc* test.

Hyperforin is able to cross the blood–brain barrier due to its lipophilic property. To evaluate the effects of Hyperforin on the improvement of CH‐mediated impairment of learning and memory in rat pups, 2.5 mg/kg hyperforin was intraperitoneally injected into the CH rat pups for 7 days before behavioral tests (Figure 8A) [46, 47]. The subjects were firstly undergone to Novel Object Recognition (NOR) experiments, which test non‐spatial memory of the rodents [48]. Although the CH pups at P21 had a lower body weight than those of the control, there were no significant changes following 7 consecutive days of hyperforin injection (Figure 8B,C). Meanwhile, the cognitive index (RI) showed no difference between the CH and hyperforin injection pups. All the three models have an RI index higher than 50%, showing exploratory preferences for new objects, as were shown by locomotor trajectory (Figure 8D–G).

**FIGURE 9:**
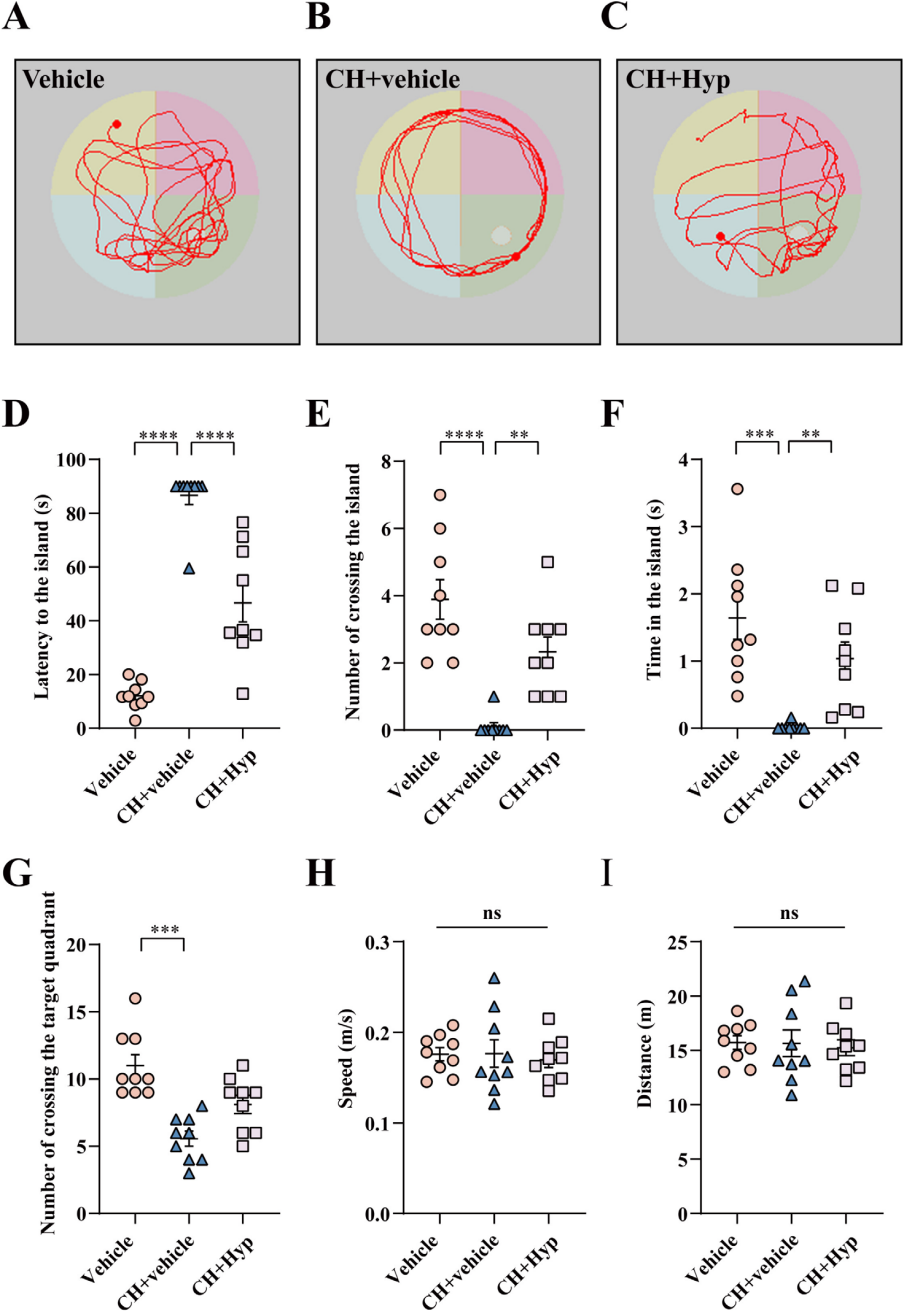
Effect of TRPC6 agonist hyperforin on cognitive function of CH pups. (A‐C) MWM experiment to test the locomotor trajectories of the rat pups. (D) Escape latency of the pups in the probing trial of MWM test. (E) Number of target island crossings in the probing trial. (F) Times staying in island in the probing trial. (G) Number of target quadrant crossings in the probing trial. (H) Average swimming speed in the probing trial. (I) Total swimming distance in the probing trial. Data are expressed as mean ± SEM, *n* = 9, *p < 0.05, **p < 0.01, ***p < 0.001, and ****p < 0.0001, oneway analysis of variance followed by Tukey's *post hoc* test or Kruskal‐Wallis test.

Next, the Morris Water Maze (MWM) was performed to assess spatial learning ability and hippocampal memory function [49]. The CH pups were seen to swim with periphery search in comparison to the multi‐direction search in the control. Following the injection of hyperforin, the swimming pattern of CH rats was partly rectified to the multi‐direction search (Figure 9A–C). The escape latency of CH subjects was markedly shortened by hyperforin (Figure 9D). In addition, the number of crossings of the island and the target quadrant, as well as the time spent on the island, was significantly increased by the agonist (Figure 9E–G). While there were no differences in the swimming speed and total distance (Figure 9H,I). The results indicate that the TRPC6 agonist is efficient in ameliorating CH‐mediated cognitive impairment of pups.

## 4 Discussion

CH in fetus and neonates will lead to neurological abnormalities and physical developmental delays [50, 51], or even cretinism [52]. The hippocampus is recognized as the primarily affected tissue by CH, characterized by dysmorphogenesis, dysfunction of neurotransmission, and, as a consequence, the cognitive deficits in the offspring [53, 54, 55]. The dendritic spines of hippocampal DGCs, which contribute to receiving excitatory inputs from the entorhinal cortex (EC) and constitute excitatory synapses [56, 57], are shown to be abnormal in the density in CH offspring. A large amount of evidence displays that Ca^2+^ and its signaling act roles in governing the development of dendrites [23, 58]. TRPC ion channels are non‐selective cation channels that manipulate neuronal calcium influx using tetramers of subunits in homo‐ and/or heteromeric configurations. They are categorized into three groups: TRPC1/TRPC4/TRPC5, TRPC2, and TRPC3/TRPC6/TRPC7 [27], among which the TRPC2 channel is a pseudogene in humans [25]. It has been shown that the TRPC1/TRPC4/TRPC5 channels are activated by receptor stimulation and involved in the manipulation of calcium pools in Ca^2+^ channels. In contrast, TRPC3, TRPC6, and TRPC7 are diacylglycerol (DAG)‐activated channels that are highly expressed in various regions of the brain [59]. So far, TRPC1, TRPC2, TRPC4, and TRPC6 channels have been found in association with the regulation of T3 [28, 29, 30], but only TRPC6 is abundantly present in the dentate gyrus of the hippocampus, reflecting its functional significance in Ca^2+^‐mediated dendritic growth [34]. In fact, dysregulation of TRPC6 activity has been found to cause many neurobiological disorders, including Alzheimer's disease [60, 61], autism spectrum disorders [62], and depression [63]. In the present study, we demonstrated that TRPC6 was involved in the regulation of CH‐mediated cognitive deficiency, suggesting multiple pathophysiological roles of the calcium channel in the development of the hippocampus.

As a calcium‐permeable channel, enforced expression of TRPC6 in neurons is shown to enhance phosphorylation of CaMKIV and CREB, as well as increase dendritic spine density and the level of presynaptic synapsin1 and postsynaptic PSD‐95 cluster [33]. Transfection of a dominant‐negative form of either CREB or CaMKIV produced the diminished effects of TRPC6‐induced spine formation [33, 34], suggesting that the CaMKIV/CREB pathway seems to be crucial for the TRPC6 in regulating the development of dendritic spines. However, TRPC6‐triggered SOCE is needed to activate CaMKII, and the autophosphorylated CaMKII in turn facilitates phosphorylation of GluR1 (subunits of AMPAR). This promotes AMPARs trafficking to the postsynaptic membrane, contributing to LTP maintenance by increasing membrane depolarization [64]. Knockdown of CaMKII in hippocampal neurons has been shown to impair SOCE, due to its effects on regulation of TRPC6‐mediated Ca^2+^ permeability [65, 66]. In the present study, we validated that the CaMKIV, rather than CaMKII, was regulated by the TRPC6 in the CH‐mediated deficiency of DGCs dendritic spines. It cannot be excluded that the CaMKII is also associated with such dysmorphogenesis during the hippocampal development.

In the NOR experiment, the RI index of hyperforin‐treated pups displayed no significant changes in comparison with the CH group. Meanwhile, the RI of all three groups was greater than 50%. It is readily explained that recognition memory in NOR experiments seems to be dependent on multiple brain regions [67, 68]. Although DG is responsible for multiple cognitive tasks including pattern separation and pattern completion [69, 70], several studies have demonstrated that hippocampal damage does not affect the preference for novel objects in the pups [71, 72]. Consistently, the knockdown of TRPC6 by delivery of shRNA AAV in the hippocampus was able to alter mouse nest building behavior and spontaneous alternation behavior, rather than the new object recognition test [73]. TRPC6 transgenic mice show an improved hippocampus‐dependent spatial memory in the Morris water maze paradigm, but no amelioration in NOR [33]. As such, it is reasonable to observe that CH‐mediated cognitive impairment is partially ameliorated by the TRPC6 activator.

The TRPC6 activator, such as AM‐0883, M085, and GSK1702934A, can directly activate TRPC6 via different mechanisms with considerably higher potency [74]. Hyperforin, the main component of St. John's wort extract, is a natural plant metabolite showing antibacterial, anticancer, and anti‐inflammatory properties, but with fewer side effects [75, 76]. Although being widely used in depression treatment [63], it also attenuates aggressive characteristics, including latency to the first attack and number of fights in isolation‐induced aggression [77]. In addition, Hyperforin is shown to overcome the memory impairments during the MWM test [78]. So, this pharmacological agent may represent an effective drug in the treatment of CH‐mediated cognitive deficiency.

In conclusion, CH of the neonates leads to downregulation of TRPC6 in hippocampal dentate gyrus neurons, which affects calcium influx and decreases activation of CaMKIV and downstream signaling, thereby causing abnormal growth of DGCs' dendritic spines and impaired cognitive function in the offspring. The TRPC6 agonist hyperforin is efficient in promoting growth of neuronal dendritic spines and ameliorating cognitive deficits of the CH pups. This study provides a new target for CH‐mediated developmental abnormality of the hippocampus in the offspring.

## Author Contributions

Study design: Y. Wu; manuscript writing and revising: Y. Wu; experiment implementation: T.L. and F.S.; data analysis: Y. Wu, J.S., T.L., F.S., L.L., P.C., Z.Z., Y.Z., H.L., G.S., and Y. Wang; data discussions: Y. Wu, J.S., T.L., F.S., L.L., P.C., Z.Z., Y.Z., H.L., G.S., and Y. Wang. All authors have approved the present version of the manuscript and have agreed to be accountable for all aspects of the work regarding questions related to the accuracy or integrity of any part of the work.

## Ethics Statement

All animal experiments were approved by the *Animal Care and Use Committee of Nantong University and the Jiangsu Province Animal Care Ethics Committee* (License No. SYXK (Su) 2020–0029).

## Consent

The authors have nothing to report.

## Conflicts of Interest

The authors declare no conflicts of interest.

## Supporting information


**Data S1.** cns70618‐sup‐0001‐Supinfo.pdf.

## Data Availability

The data that support the findings of this study are available from the corresponding author upon reasonable request.
