# Supplementary material for: Congenital Hypothyroidism Dysregulates TRPC6 to Mediate Abnormal Dendritic Spine Growth of Hippocampal Neurons
Source: CNS Neurosci Ther. 2025 Sep 25;31(9):e70618. doi: 10.1111/cns.70618 (PMC12461174; doi:10.1111/cns.70618)

Figure6

Figure6B Full unedited gel/blot for CaMKIV

CaMKIV-1

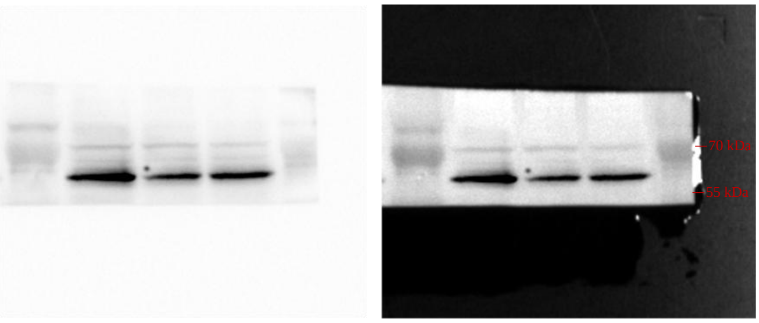

GAPDH-1

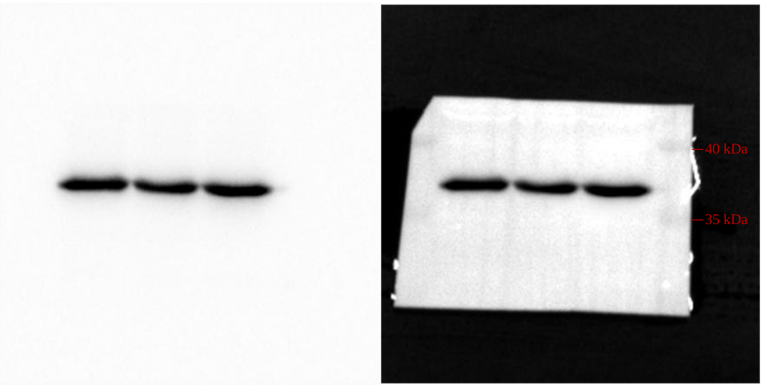

CaMKIV-2

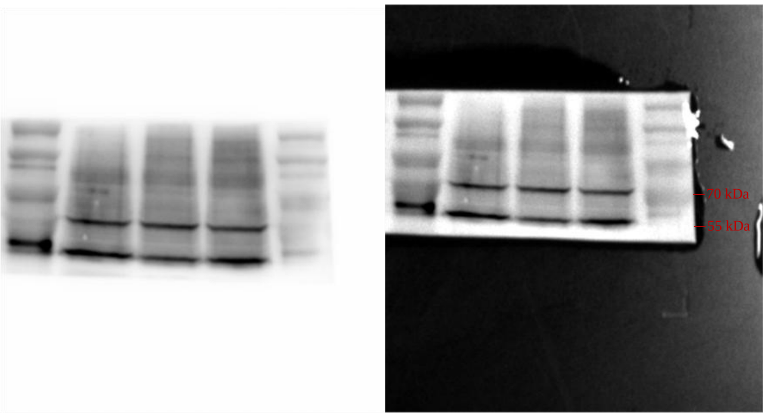

GAPDH-2

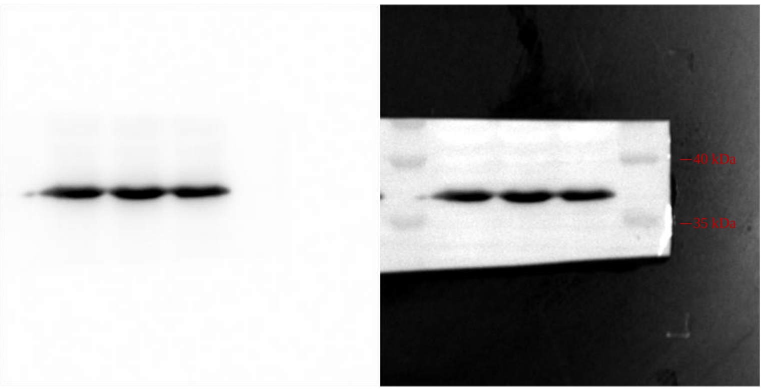

CaMKIV-3

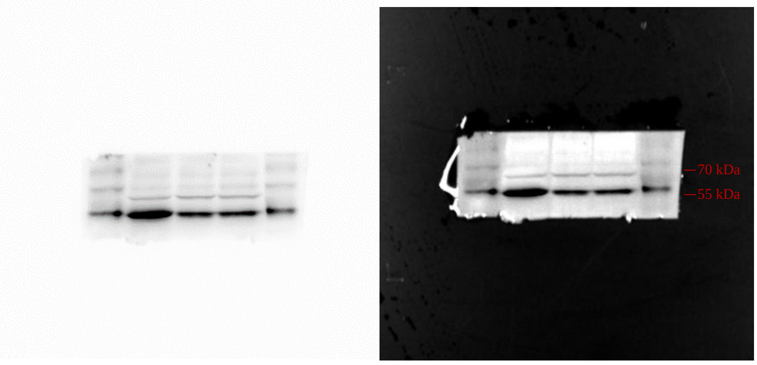

GAPDH-3

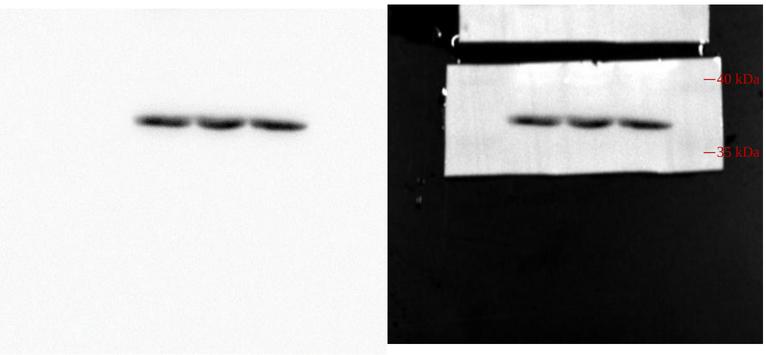

CaMKIV-4

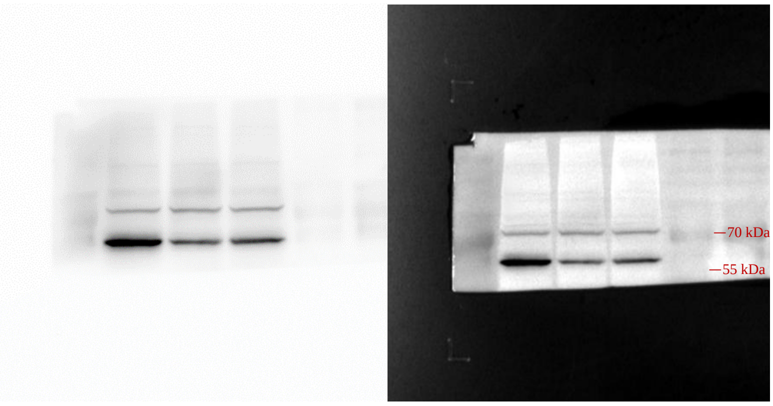

GAPDH-4

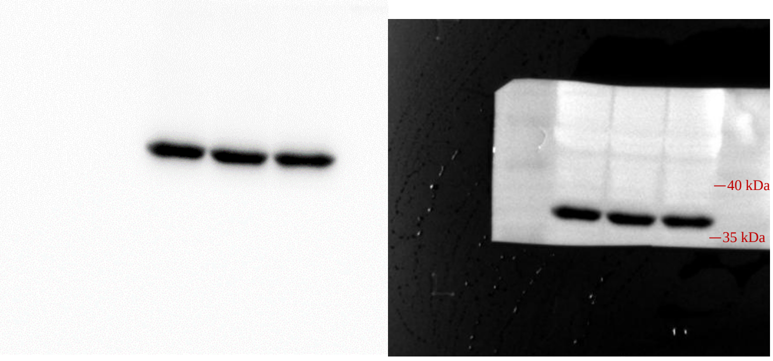

Figure6 C Full unedited gel/blot for Egr3

Egr3-1

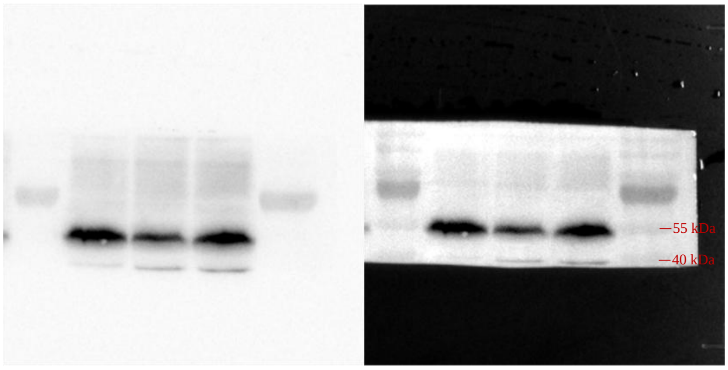

GAPDH-1

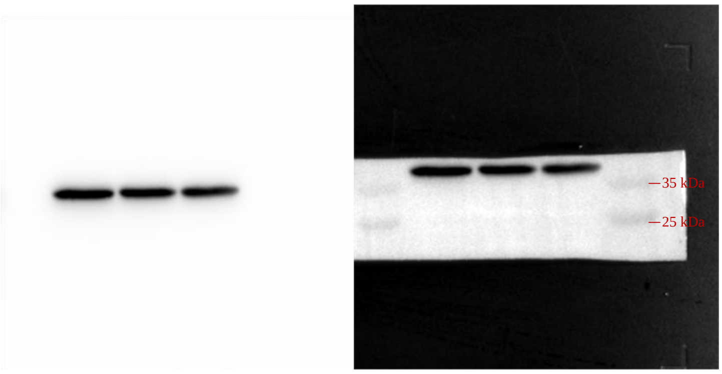

Egr3-2

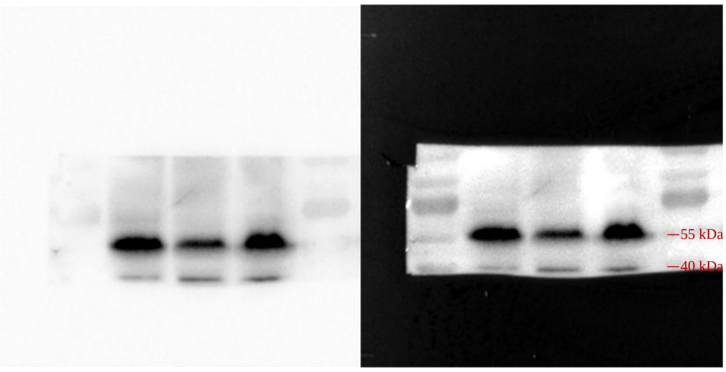

GAPDH-2

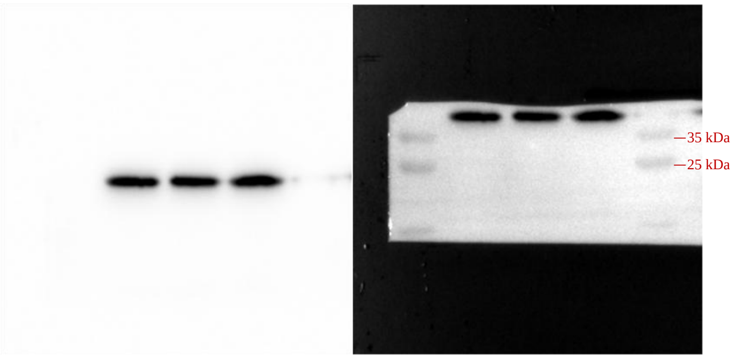

Egr3-3

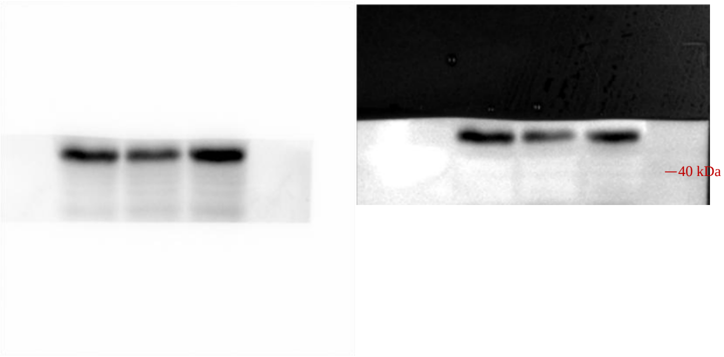

GAPDH-3

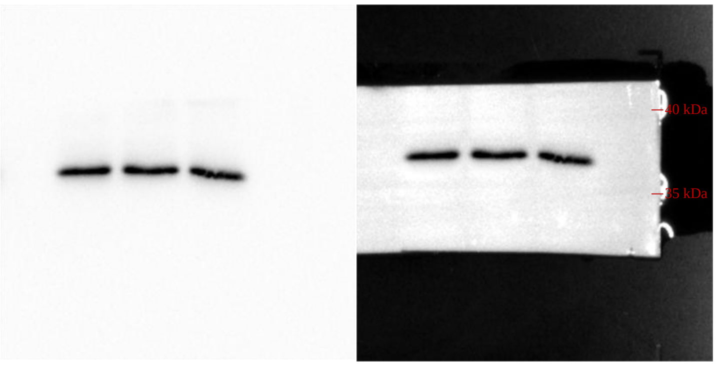

Figure6 D Full unedited gel/blot for BDNF

BDNF-1

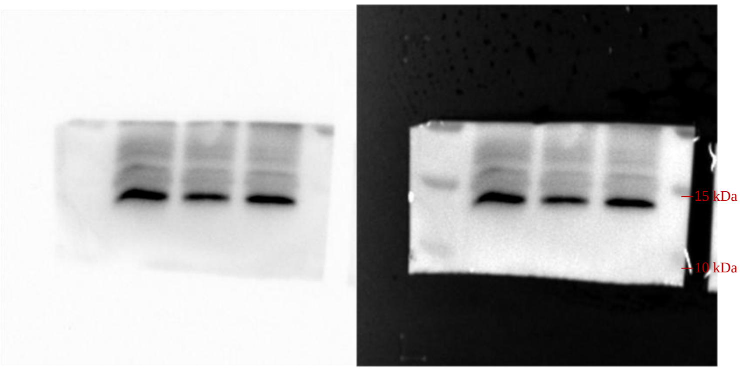

GAPDH-1

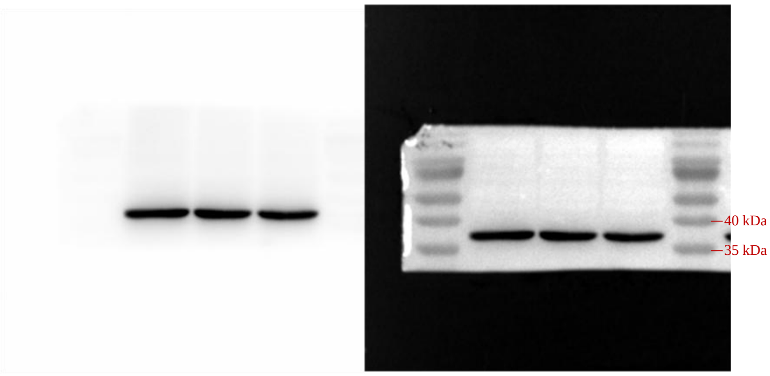

BDNF-2

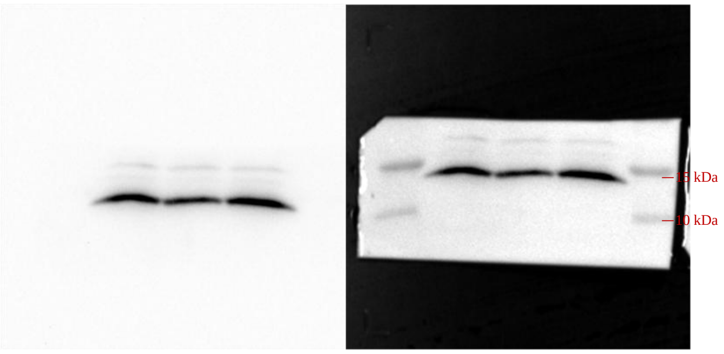

GAPDH-2

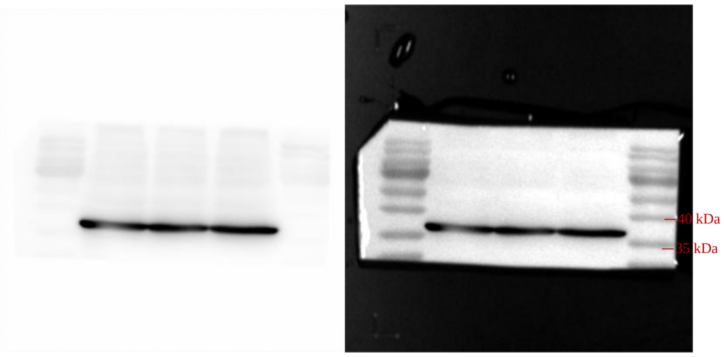

BDNF-3

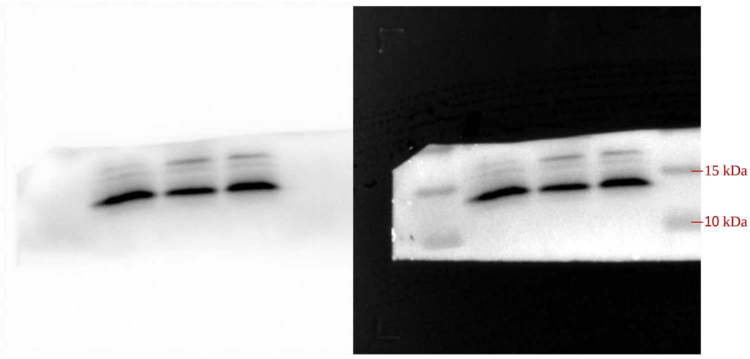

GAPDH-3

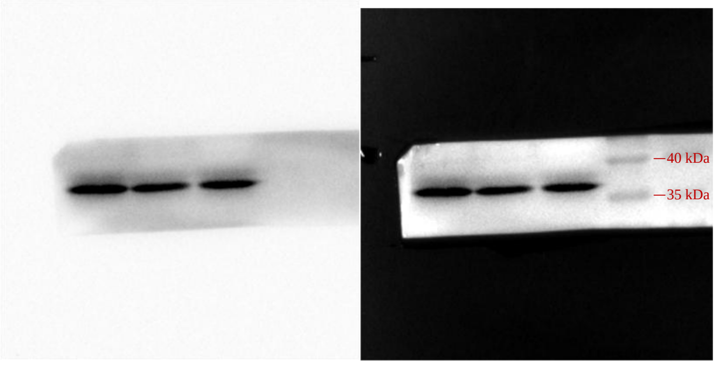

Figure6 E Full unedited gel/blot for CREB

CREB-1

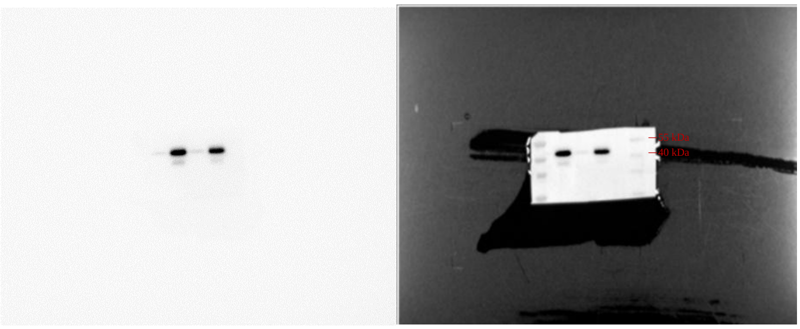

Lamin B1-1

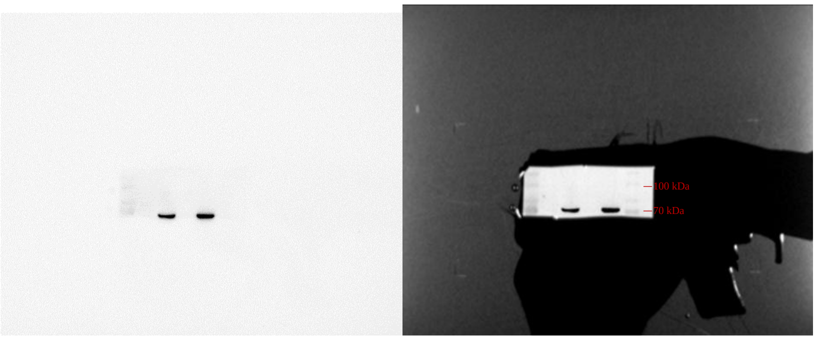

GAPDH-1

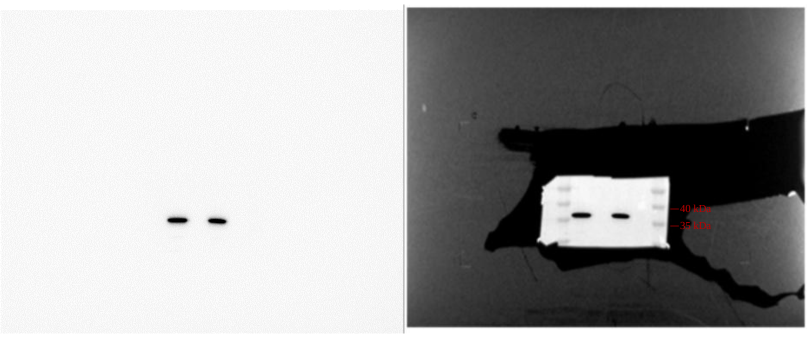

CREB-2

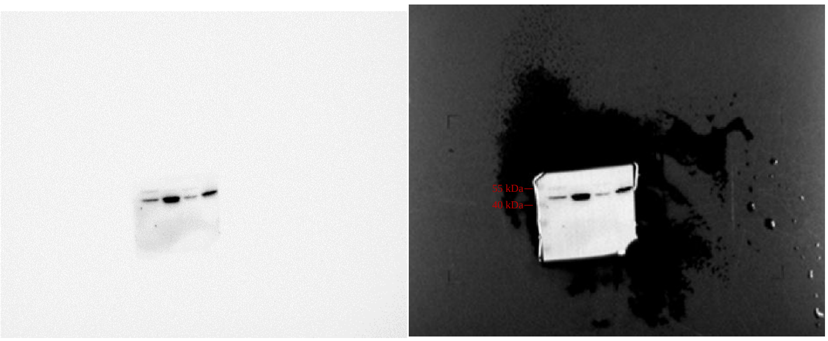

Lamin B1-2

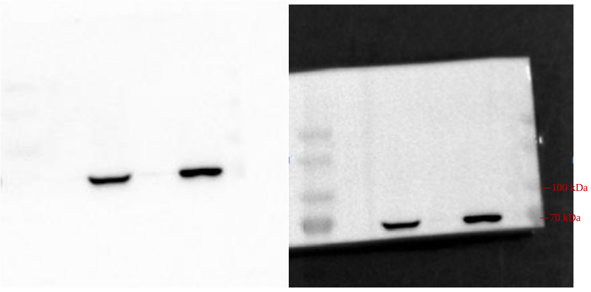

GAPDH-2

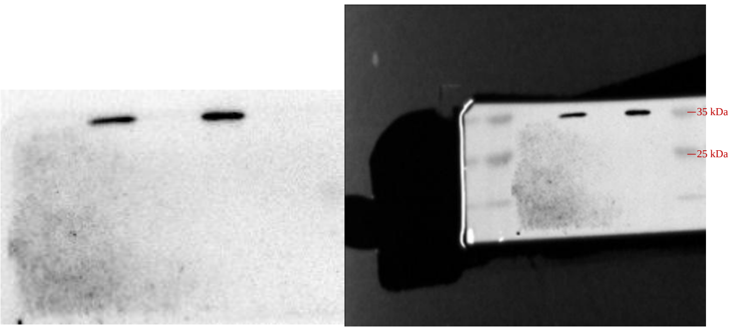

CREB-3

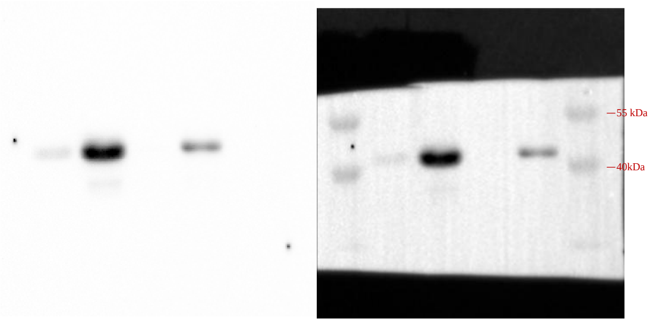

Lamin B1-3

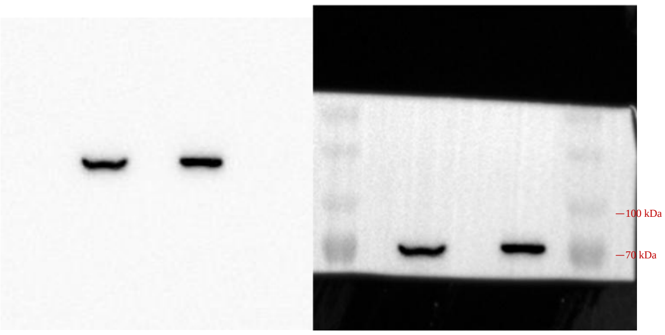

GAPDH-3

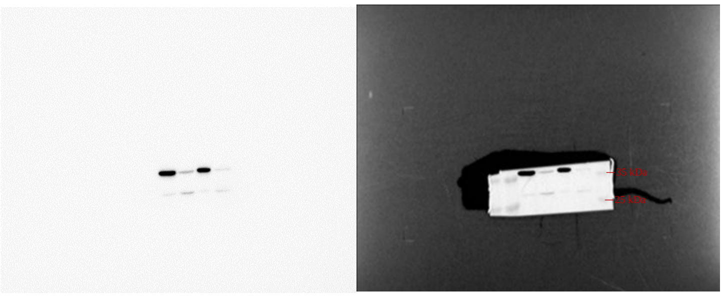

Supplement: Supplementary file 1 — Data S1. cns70618‐sup‐0001‐Supinfo.pdf. [file CNS-31-e70618-s001.pdf]
